# Analysis of Expression, Cellular Localization, and Function of Three Inhibitors of Apoptosis (IAPs) from *Litopenaeus vannamei* during WSSV Infection and in Regulation of Antimicrobial Peptide Genes (AMPs)

**DOI:** 10.1371/journal.pone.0072592

**Published:** 2013-08-14

**Authors:** Pei-Hui Wang, Ding-Hui Wan, Zhi-Hua Gu, Wei Qiu, Yong-Gui Chen, Shao-Ping Weng, Xiao-Qiang Yu, Jian-Guo He

**Affiliations:** 1 MOE Key Laboratory of Aquatic Product Safety/State Key Laboratory of Biocontrol, School of Life Sciences, Sun Yat-Sen University, Guangzhou, People’s Republic of China; 2 School of Marine Sciences, Sun Yat-Sen University, Guangzhou, People’s Republic of China; 3 Division of Cell Biology and Biophysics, School of Biological Sciences, University of Missouri-Kansas City, Kansas City, Missouri, United States of America; Chang Gung University, Taiwan

## Abstract

Inhibitors of apoptosis (IAPs) play important roles in apoptosis and NF-κB activation. In this study, we cloned and characterized three IAPs (LvIAP1-3) from the Pacific white shrimp, *Litopenaeusvannamei* . LvIAP1-3 proteins shared signature domains and exhibited significant similarities with other IAP family proteins. The tissue distributions of LvIAP1-3 were studied. The expression of *LvIAP1-3* was induced in the muscle after white spot syndrome virus (WSSV) infection. *LvIAP1* expression in the gill, hemocytes, hepatopancreas, and intestine was responsive to WSSV and *Vibrio* *alginolyticus* infections. *LvIAP2* expression in the gill, hemocytes, and hepatopancreas was also responsive to WSSV infection. The expression of *LvIAP3* in the gill, hemocytes, and intestine was reduced after *V* *. alginolyticus* infection. When overexpressed in *Drosophila* S2 cells, GFP labeled-LvIAP2 was distributed in the cytoplasm and appeared as speck-like aggregates in the nucleus. Both LvIAP1 and LvIAP3 were widely distributed throughout the cytoplasm and nucleus. The expression of *LvIAP1*, *LvIAP2*, and *LvIAP3* was significantly knocked down by dsRNA-mediated gene silencing. In the gill of *LvIAP1*- or *LvIAP3*-silenced shrimp, the expression of WSSV *VP28* was significantly higher than that of the dsGFP control group, suggesting that LvIAP1 and LvIAP3 may play protective roles in host defense against WSSV infection. Intriguingly, the *LvIAP2*-silenced shrimp all died within 48 hours after dsLvIAP2 injection. In the hemocytes of *LvIAP2*-silenced shrimps, the expression of antimicrobial peptide genes (AMPs), including *Penaeidins, lysozyme, crustins,* *Vibrio* *penaeicidae-induced* *cysteine and proline-rich peptides* (*VICPs*), was significantly downregulated, while the expression of anti-lipopolysaccharide factors (ALFs) was upregulated. Moreover, LvIAP2 activated the promoters of the NF-κB pathway-controlled AMPs, such as shrimp *Penaeidins* and *Drosophila drosomycin* and *attacin A*, in *Drosophila* S2 cells. Taken together, these results reveal that LvIAP1 and LvIAP3 might participate in the host defense against WSSV infection, and LvIAP2 might be involved in the regulation of shrimp AMPs.

## Introduction

Apoptosis is a genetically programmed process of controlled cell suicide that plays critical roles in organismal development, homeostasis, and the immune system through elimination of cells that are no longer useful [1]. The dysregulation of apoptosis contributes to the pathogenesis of various diseases, such as cancers and autoimmunity [2,3]. Because of its destructive effect on living cells, apoptosis is tightly controlled by multiple regulators [4]. Inhibitors of apoptosis proteins (IAPs) inhibit the activity of caspases, the main executors of the apoptosis process, and play important roles in regulating the progression of apoptosis from insects to humans [4,5]. IAP was first identified as a baculovirus gene that inhibits apoptosis in virus-infected *Spodoptera* *frugiperda* insect cells to enhance viral multiplication [5]. Since then, many IAP homologs have been identified in yeast, nematodes, flies, and mammals [5]. There are four and eight members of the IAP family in *Drosophila* and humans, respectively [5]. The IAP family proteins are characterized by the presence of one to three N-terminal zinc-binding baculoviral IAP repeat (BIR) domains [6]. These BIR domains bind directly to the caspases and inhibit their activities. Therefore, BIR domains are essential for the anti-apoptotic properties of IAPs [6]. Some IAPs also contain a C-terminal RING domain, which has ubiquitin E3 ligase activity [6]. The RING domain ubiquitinates the proteins that bind to IAPs, including caspases and IAPs themselves [6]. The ubiquitinated caspases are inactivated and the ubiquitinated IAPs are subjected to proteasome degradation [6].

In addition, IAPs also play important roles in immune signaling regulation from insects to mammals [6–8]. In *Drosophila* , the Toll and immune deficiency (IMD) pathways are the major regulators of the immune responses [9–11]. Gram-positive bacteria and fungi activate the Toll pathway, while Gram-negative bacterial infections activate the IMD pathway [9–11]. Activation of the Toll and IMD pathways initiates an intracellular signaling cascade to activate the NF-κB family proteins Dorsal/Dif and Relish, respectively, promoting the expression of immune-related genes, such as antimicrobial peptide genes (AMPs) [9–11]. Although no components of *Drosophila* Toll and IMD pathways have been identified as detectors for viral infections, viruses activate both pathways, which contribute to the restriction of viral replication [12–14]. Knock-down of *Drosophila* IAP2 (DIAP2) in insect cells reduced the expression of AMPs induced by Gram-negative bacteria, suggesting a role of DIAP2 in the IMD pathway [15–18]. The RNAi-mediated silencing of DIAP2 in the adult fat body also abrogated AMP gene expression induced by Gram-negative bacterial infection. Importantly, DIAP2 null flies exposed to Gram-negative bacteria did not activate the IMD pathway and died [15–18]. The exogenous expression of wild-type DIAP2, but not the E3-deficient RING mutant, rescued the DIAP2 null flies [16,18]. DIAP2 is required to sustain AMP expression in *Drosophila* S2 cells. However, the role of DIAP2 is limited to the IMD signaling, as DIAP2 null flies showed no defects in immune responses triggered via the Toll pathway, such as response against fungal infections [15–18]. Recently, it was reported that Gram-negative bacterial infections induce binding of DIAP2 to the caspase8 homolog DREDD, targeting it for polyubiquitination in a RING finger-dependent manner for Relish processing and subsequent AMP expression [19,20]. The functions of IAPs in defense against microbial infection and induction of NF-κB activity in mammals are evolutionarily conserved [7,8,21–24]. For example, mammalian cIAP-1, cIAP-2, and XIAP expressions are induced by NF-κB and contribute to NF-κB-mediated protection of some cells against TNFα-induced apoptosis [7,22,24]. However, the function of IAP2 in NF-κB activation is still unclear in other invertebrates, except *Drosophila* .

Infections with white spot syndrome virus (WSSV), one of the most common and destructive pathogens in shrimp aquaculture, result in 100% mortality of penaeid shrimp within 3-10 days. Both apoptosis and shrimp AMPs, including Penaeidins (PENs), crustins, and anti-lipopolysaccharide factors (ALFs), are important in the defense against WSSV infection [25–35]. Apoptosis induced by WSSV infection is important for successful WSSV pathogenesis [25–27,31]. To manipulate host apoptosis, WSSV modulates the expression of shrimp apoptosis-related genes, such as *PmCasp*, *PjCaspase*, *Pm-fortilin* and *VDAC*, to actively promote apoptosis to spread virus progeny to neighboring cells; in contrast, WSSV also encodes two anti-apoptosis proteins, AAP-1 (ORF390 or WSSV449) and WSSV222, to block apoptosis in order to prevent premature host cell death and maximize virus progeny [27–29,36–40]. The function of one shrimp IAP in regulating hemocyte apoptosis has been studied [41,42]; however, the roles of shrimp IAPs in defending against WSSV infection and regulating AMP expression through the NF-κB pathway remain unknown [41,42]. In this study, we cloned three IAPs from the model crustacean *Litopenaeusvannamei* and investigated the roles of these proteins during WSSV infection and in regulation of shrimp AMP expression.

## Materials and Methods

### 2.1: Shrimp culture

Healthy Pacific white shrimp ( *L* *. vannamei* ), approximately 4-5 g in body weight, were obtained for gene cloning and tissue distribution analysis, and those approximately 1-2 g in body weight were used for dsRNA-mediated gene silencing. All shrimp were purchased from a local shrimp farm in Zhuhai, Guangdong Province, China. The shrimp were cultured in a recirculating water tank system containing air-pumped seawater (2.5% salinity) at 24-26 °C and fed commercial feed at 5% of body weight twice a day, as previously described [43]. The shrimp were cultured for at least seven days to facilitate acclimation before the experiments were conducted.

### 2.2: RNA extraction and cDNA synthesis

Total RNA was extracted from the gill of *L* *. vannamei* using an RNeasy Mini Kit (Qiagen, Germany). Residual genomic DNA was removed using RNase-free DNase I (Qiagen, Germany). The cDNA template for rapid amplification of cDNA ends (RACE) PCR was prepared using a SMARTer™ RACE cDNA Amplification Kit (Clontech, USA). For gene cloning, first-strand cDNA was prepared using a PrimeScript™ 1st strand cDNA Synthesis Kit (Takara Bio, China). For the real-time quantitative PCR (qPCR) analysis, first-strand cDNA was prepared using a PrimeScript™ RT Reagent Kit (Takara Bio, China).

### 2.3: Rapid amplification of cDNA ends (RACE)

EST (expression sequence tag) sequences showing similarities to IAPs were identified using the NCBI EST database of *L* *. vannamei* . Based on the ESTs, we designed gene-specific primers (GSPs; listed in Table 1). The full-length cDNA of LvIAP1-3 were obtained using 5′- and 3'-RACE approach, as described in our previous studies [44–46].

**Table 1:** PCR primers used in this study.

**Primer**		**Primer sequence (5’–3’)**
**cDNA cloning**		
LvIAP1-5’ RACE1		CATCCTATTGCCAGTTCATCC
LvIAP1-5’RACE2		TGACCTTGTCCGTTGCTTTG
LvIAP1-3’ RACE1		GAAGATGGCTGCTGCTGG
LvIAP1-3’RACE2		TGACTTGGAAATGTACCGACAG
LvIAP2-5’ RACE1		CGTCATCTCCTTTCTTCGTGTA
LvIAP2-5’ RACE2		TCTCGTACCTCAGGCTATCGTA
LvIAP2-3’ RACE1		ATGCCGAGATGGATGTTGTG
LvIAP2-3’ RACE2		CTCAGTGCCCCATCTGTAGGA
LvIAP3-5’ RACE1		TCATTCCTAAAGTCAATCGTGCT
LvIAP3-5’ RACE2		CTAAGTCATCAGGGGATAACCAAT
LvIAP3-3’ RACE1		TGTTAATGAGCCAGATAGCACG
LvIAP3-3’ RACE2		GCCACGTTACATTTTGTAGGTCAG
**qPCR analysis**		
qPCRLvIAP1-F		GAGATGAGCACAGAGGAAAAGAG
qPCRLvIAP1-R		ATGGATGAACTGGCAATAGGA
qPCRLvIAP2-F		CCCGCACTGTCCATTTATCA
qPCRLvIAP2-R		GCCTTGACGTTCCACATTCA
qPCRLvIAP3-F		GGAACATACCTTTGGTTAGGAGTC
qPCRLvIAP3-R		TCAATCGTGCTATCTGGCTCA
qPCRLvEF-1α-F		GAAGTAGCCGCCCTGGTTG
qPCRLvEF-1α-R		CGGTTAGCCTTGGGGTTGAG
dsRNA preparation*		
dsGFP-F		AGTGCTTCAGCCGCTACCC
dsGFP-R		GCGCTTCTCGTTGGGGTC
dsGFP(T7)-F		T A A T A C G A C T C A C T A T A G GAGTGCTTCAGCCGCTACCC
dsGFP(T7)-R		T A A T A C G A C T C A C T A T A G GGCGCTTCTCGTTGGGGTC
DsLvIAP1-F		AGCAAGGGTTTGGAGAACTTCT
dsLvIAP1-R		ATGACAAAGGATAAAGAAAGAGAGG
dsLvIAP1(T7)-F		T A A T A C G A C T C A C T A T A G GAGCAAGGGTTTGGAGAACTTCT
dsLvIAP1(T7)-R		T A A T A C G A C T C A C T A T A G GATGACAAAGGATAAAGAAAGAGAGG
dsLvIAP2-F		CGCTTGGTAGACAGGCTAAGAT
dsLvIAP2-R		TACACGAAGAAAGGAGATGACG
dsLvIAP2(T7)-F		T A A T A C G A C T C A C T A T A G GCGCTTGGTAGACAGGCTAAGAT
dsLvIAP2(T7)-R		T A A T A C G A C T C A C T A T A G GTACACGAAGAAAGGAGATGACG
dsLvIAP3-F		ATCACCTGTCTCCCATTTACCT
dsLvIAP3-R		TCATGAAGTGGGAGAAGGGTAA
dsLvIAP3(T7)-F		T A A T A C G A C T C A C T A T A G GATCACCTGTCTCCCATTTACCT
dsLvIAP3(T7)-R		T A A T A C G A C T C A C T A T A G GTCATGAAGTGGGAGAAGGGTAA
protein expression**		
pA5.1LvIAP1-F		CGGGGTACCATGACAAAGGATAAAGAAAGAGAGG
pA5.1LvIAP1-R		GCTCTAGAAGCAAGGGTTTGGAGAACTTCT
pA5.1LvIAP2-F		AAGGAAAAAAGCGGCCGCCGCCACCATGGGTGATATGTC-CCACGATC
pA5.1LvIAP2-F		GCTCTAGAGGAGACAATAGGTTTGATGGTGAAT
pA5.1LvIAP3-F		CGGGGTACCATGGCTCTATTAGATGACCATATGG
pA5.1LvIAP3-F		GCTCTAGACTTTGGAATATTACCAACTGGTTTTC

* T7 RNA polymerase binding site is underlined; ** primers used in the cellular localization and luciferase reporter assays were the same.

### 2.4: Cloning of novel *L*. *vannamei* AMPs

To examine the regulation of *L* *. vannamei* AMPs through *LvIAP2*, novel shrimp AMPs, including *Lvlysozyme*, *Lvcrustin1-3*, *LvVICP1-2*, and *LvALF1-3*, were cloned according to the EST sequences in NCBI database or using degenerated primers as previously described [44–47].

### 2.5: Amino acid sequence analysis

ScanProsite (http://prosite.expasy.org/scanprosite/) and a simple modular architecture research tool (SMART, http://smart.embl-heidelberg.de) were used to predict the domain structure of LvIAP1-3. Multiple sequence alignments were performed using the ClustalX 2.0 program. Neighbor-joining (NJ) phylogenic trees were constructed using MEGA 4.0 software (http://www.megasoftware.net/). The bootstrap sampling was repeated 1,000 times.

### 2.6: Real-time qPCR analysis

Gram-negative *Vibrio* *alginolyticus* and WSSV inocula were prepared and quantified, as described in previous studies [45,48]. In the microbial challenge experiments, each *L* *. vannamei* was injected intramuscularly at the third abdominal segment with 100 µl of *V* *. alginolyticus* inoculum (approximately 7 × 10^6^ CFU/shrimp) or with 100 µl of WSSV inoculum (approximately 10^7^ copies/shrimp). Phosphate-buffered saline (PBS)-injected shrimp were used as controls. At 0, 3, 6, 12, 24, 36, 48, and 72 hours post-injection (hpi), five shrimp from each group were randomly selected to harvest the gill, hemocytes, intestine, hepatopancreas, and muscle. Healthy *L* *. vannamei* tissues, including the hemocytes, eyestalk, gill, heart, hepatopancreas, stomach, intestine, nerve, muscle, pyloric cecum, and epithelium were collected for the tissue distribution analysis. Total RNA isolation and first-strand cDNA preparation were described in Section 2.2. The expressions of *LvIAP1-3*,WSSV *VP28*, *L. vannamei* *AMPs* (including *LvPEN2*, *LvPEN3*, *LvPEN4*, *Lvlysozyme*, *Lvcrustin1*, *Lvcrustin2*, *Lvcrustin3*, *LvVICP1*, *LvVICP2*, *LvALF1*, *LvALF2*, and *LvALF3*) were measured using *qPCR* by the relative standard curve method for calculation of changes in gene expression as described in previous studies [44,45]. The expression of *L* *. vannamei* elongation factor 1α (*LvEF-1α*) was used as the internal control. Three replicate qPCRs were performed and three shrimp were analyzed per sample. The mRNA expression level in the untreated group (0 hpi) was set as 1.0. The standard curves for *LvIAP1-3* and *LvEF-1α* were generated through triplicate reactions of serially 10-fold dilutions (i.e., 10 different cDNA concentrations). The efficiencies for *LvIAP1*, *LvIAP2*, *LvIAP3*, and *LvEF-1α* were 1.926, 1.940, 1.953, and 1.953, respectively.

### 2.7: Plasmid construction

To express LvIAP1-3 in *Drosophila* S2 cells for cellular localization and functional studies, the pAc5.1-LvIAP1-3 vectors were constructed using the pAc5.1/V5-His A vector (Invitrogen, USA) as previously described [44,47]. We constructed an expression plasmid, pAc5.1-N-GFP, which efficiently expresses green fluorescent protein (GFP) in *Drosophila* S2 cells, as described in our previous studies [44,45]. The complete *LvIAP1-3* open reading frames (ORFs) were inserted into the pAc5.1-N-GFP vector to create the pAc5.1-LvIAP1-3-GFP, expressing full-length LvIAP1-3 fused with GFP. The luciferase reporter vectors, including pGL3-PEN453, pGL3-PEN309, pGL3-PEN4, pGL3-Drs, pGL3-AttA, pGL3-WSSV069, pGL3-WSSV303, and pGL3-WSSV371, had been constructed in previous studies [44,47] and were predominantly regulated through NF-κB activation [45,47,49–52]. The promoter sequences of PEN453, PEN309, PEN4, Drs, AttA, WSSV069, WSSV303, and WSSV371 are provided in Figure S1.

### 2.8: Cell culture

Because no immortalized shrimp cell line is currently available, *Drosophila Schneider* 2 (S2) cells (Invitrogen), derived from a macrophage-like lineage, were used to analyze the cellular localization and function of LvIAP1-3. *Drosophila* S2 cells were maintained at 28 °C in Schneider’s *Drosophila* *medium* (SDM) (Invitrogen) without CO_2_ and supplemented with 10% fetal bovine serum (FBS). When the culture density reached 6-20 × 10^6^ viable cells ml^−1^, the *Drosophila* S2 cells were passaged onto a new plate at a density of 5 × 10^5^ viable cells ml^−1^.

### 2.9: Cellular localization analysis

*Drosophila* S2 cells were seeded onto poly-l-lysine-treated coverslips in 24-well plates at 24 hours before transfection. pAc5.1-LvIAP1-3-GFP were transfected into *Drosophila* S2 cells using Effectene Transfection Reagent (Qiagen, Germany) according to the manufacturer’s protocol. Thirty-six hours after transfection, the cells on the coverslips were washed twice with PBS, fixed in Immunol Staining Fix Solution (Beyotime, China) and stained with Hoechst 33258 (Beyotime, China). The coverslips were subsequently examined for protein cellular localization using a Leica laser scanning confocal microscope as previously described [45–47].

### 2.10: Dual luciferase reporter assays

*Drosophila* S2 cells were seeded onto a 96-well culture plates in 100 µl medium at 2 × 10^5^ cells ml^−1^ for 24 hours prior to transfection. To examine whether LvIAP2 affects the promoter activities of NF-κB-controlled AMPs, the expression vector pAC5.1-LvIAP2 (0.05 µg per well) was cotransfected with the luciferase reporter gene pGL3-Basic, pGL3-PEN453, pGL3-PEN309, pGL3-PEN4, pGL3-Drs or pGL3-AttA (0.05 µg per well) as described in our previous studies [44,47]. The pRL-TK *Renilla* luciferase vector was used as an internal control. The cells were harvested and lysed at 36 hours after transfection to examine luciferase activities using the Dual-Luciferase Reporter Assay System (Promega, USA).

### 2.11: Preparation of dsRNA and gene silencing through dsRNA injection in vivo

The double-stranded RNAs (dsRNAs) of *LvIAP1-3* and *GFP* (were prepared using T7 RiboMAX Express Kit (Promega, USA) as previous described [43]. Briefly, DNA templates for the production of dsLvIAP1-3 and dsGFP were PCR amplified using gene-specific primers with the T7 RNA polymerase binding site at the 5’ terminus to produce sense and anti-sense RNA strands separately. The single-stranded RNA was annealed to generate dsRNA. After purification, the dsRNA was quantified and stored at -80° C. For the dsRNA-mediated gene silencing experiments, the experimental group (1-2 g per shrimp) was treated with dsLvIAP1, dsLvIAP2 or dsLvIAP3 (1 µg/g shrimp) through intramuscular injection, while the control groups were injected with dsGFP and PBS, respectively. To determine the silencing effects, the gill samples from at least three shrimp from each treatment were collected at 0, 24, 72, 120, and 144 hours post-dsRNA injection (hpi), and the total RNA was extracted. The total RNA from the gills of dsRNA-injected *L* *. vannamei* was reverse-transcribed into the first-strand cDNA to assess the gene silence efficiency. For LvIAP2, the hemocytes were collected to assess the gene silencing efficiency because we did not observe an obvious reduction in gene expression in the gill of dsLvIAP2-injected shrimps.

### 2. 12. The expression level of endogenous *L*. *vannameiv* AMPs in dsLvIAP2-injected shrimp

The expression levels of *L. vannamei* *PENs*, *lysozyme, crustins*, *VICPs*, and *ALFs* (*LvPEN2*, *LvPEN3*, *LvPNE4, Lvlysozyme, Lvcrustin1, Lvcrustin2, Lvcrustin3, LvVICP1, LvVICP2, LvALF1, LvALF2*, and *LvALF3*) were detected using the cDNA templates prepared from the hemocytes of dsLvIAP2-injected shrimps by qPCR as described in Section 2.5.

### 2. 13: The WSSV infection experiments in dsRNA-injected *L*. *vannamei*

The efficiency of gene silencing in dsLvIAP1- and dsLvIAP3-injected *L* *. vannamei* was significant compared with that of the control groups (> 80%) at all examined time points selected for qPCR analysis. In the WSSV infection experiments, *L* *. vannamei* were intramuscularly infected with a WSSV inoculum (approximately 10^7^ copies/shrimp) at 48 hours after dsRNA injection and the gills were collected at 0, 3, 6, 12, 24, 36, and 48 hpi to assess WSSV *VP 28* expression.

### 2.14: Statistical analyses

The data are presented as the means ± standard error of the mean (SEM). Student’s t-test was used to compare the means of two samples using Microsoft Excel. The chi-square statistical analysis was performed to assess differences in the mortality rates through a comparison of the mortality of the dsLvIAP2 injection group with that of the PBS or dsGFP-injected group. In all cases, the differences were considered statistically significant at p < 0.05 and highly significant at p < 0.01.

## Results

### 3.1: Cloning and sequence analysis of LvIAP1-3, Lvlysozyme, Lvcrustin1-3, LvALF1-3, and LvVICP1-2

**Figure 1:**
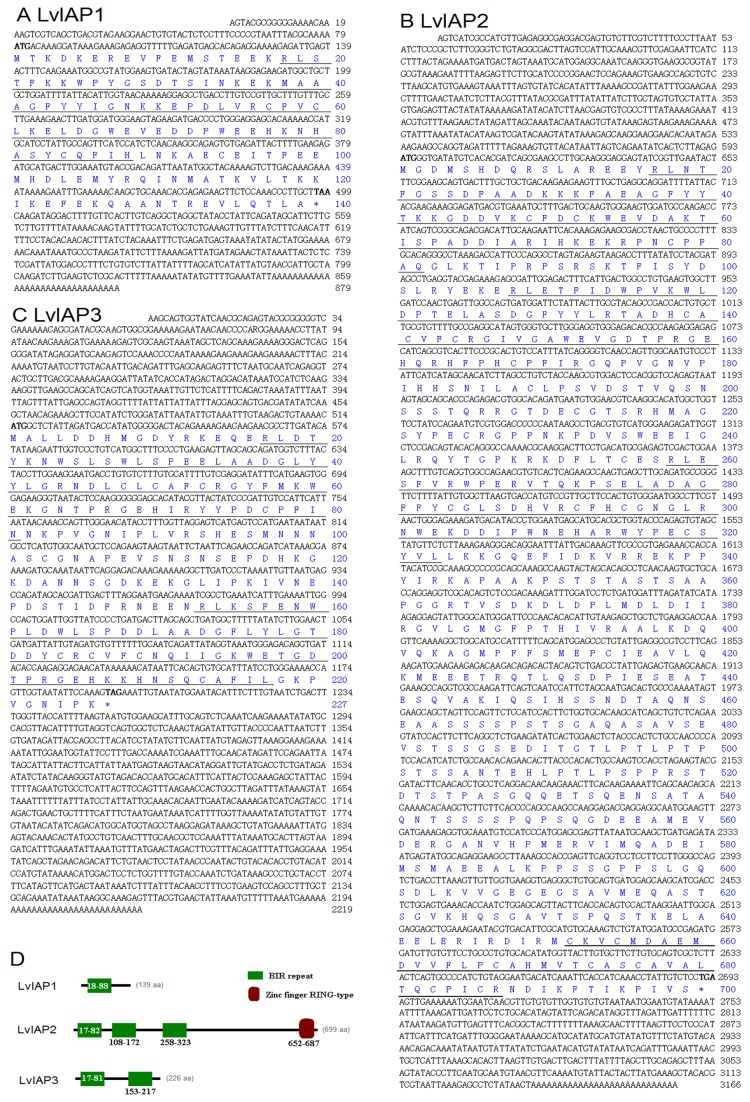
Nucleotide and deduced amino acid sequences of LvIAP1 (A), LvIAP2 (B), and LvIAP3 (C) from *L* *. vannamei* . The full-length cDNA (upper row) and deduced amino acid (lower row) sequences of LvIAP1-3 are shown. The initiation (ATG) and stop (TAA, TGA or TAG) codons are bolded. The BIR repeat motifs of LvIAP1-3 and the zinc finger RING-type domain of LvIAP3 are underlined. (D) Domain architecture of LvIAP1-3. The full-length amino acid sequences of LvIAP1-3 were subjected to analysis using ScanProsite (http://prosite.expasy.org/scanprosite/) and the simple modular architecture research tool (SMART, http://smart.embl-heidelberg.de) to predict the domain structure of LvIAP1-3. The green box indicates the BIR repeat motif, and the brown box represents the zinc finger RING-type domain.

Based on the EST sequences of *L* *. vannamei* in the NCBI database, the full-length cDNAs of three novel *L* *. vannamei* IAPs (LvIAP1-3) were cloned. *LvIAP1* cDNA was 879 bp with an ORF of 420 bp, encoding a putative protein of 139 amino acids, a 5’ untranslated region of 79 bp, and a 3’ untranslated region of 380 bp (Figure 1A). *LvIAP2* cDNA was 3, 166 bp with an ORF of 2,100 bp, encoding a putative protein of 699 amino acids, a 5’ untranslated region of 593 bp, and a 3’ untranslated region of 473 bp (Figure 1B). *LvIAP3* cDNA was 2, 219 bp with an ORF of 1,176 bp, encoding a putative protein of 496 amino acids, a 5’ untranslated region of 59 bp, and a 3’ untranslated region of 399 bp (Figure 1C).

LvIAP1 contains one BIR domain and shares 38% and 29% identities to human survivin and *Drosophila* Deterin, respectively (Figure 1D). LvIAP2 contains three BIR domains and a C-terminal RING domain, and is 27% and 30% identical to *Drosophila* IAP1 and IAP2, respectively (Figure 1D). LvIAP3 is a completely novel member of the IAP family proteins, possessing two BIR domains, and it is not similar to any known IAPs (Figure 1D).

To investigate the regulation of shrimp AMPs through LvIAP2 *in vivo*, one novel *Lvlysozyme*, three novel *Lvcrustins*, three novel *LvALFs*, and two novel *LvVICPs* were cloned. *LvVICPs* ( *Vibrio* *penaeicidae* -induced cysteine and proline-rich peptides) are homologs of *Stylicins* in Paciﬁc blue shrimp *Litopenaeusstylirostris* , which are new members of the recently identified shrimp AMPs with strong antifungal activity against *Fusarium oxysporum*, a pathogenic fungus of shrimp. The sequences of *Lvlysozyme*, *Lvcrustin1-3*, *LvALF1-3*, and *LvVICP1-2* are provided in Figure S2.

### 3.2: Phylogenetic tree construction

**Figure 2:**
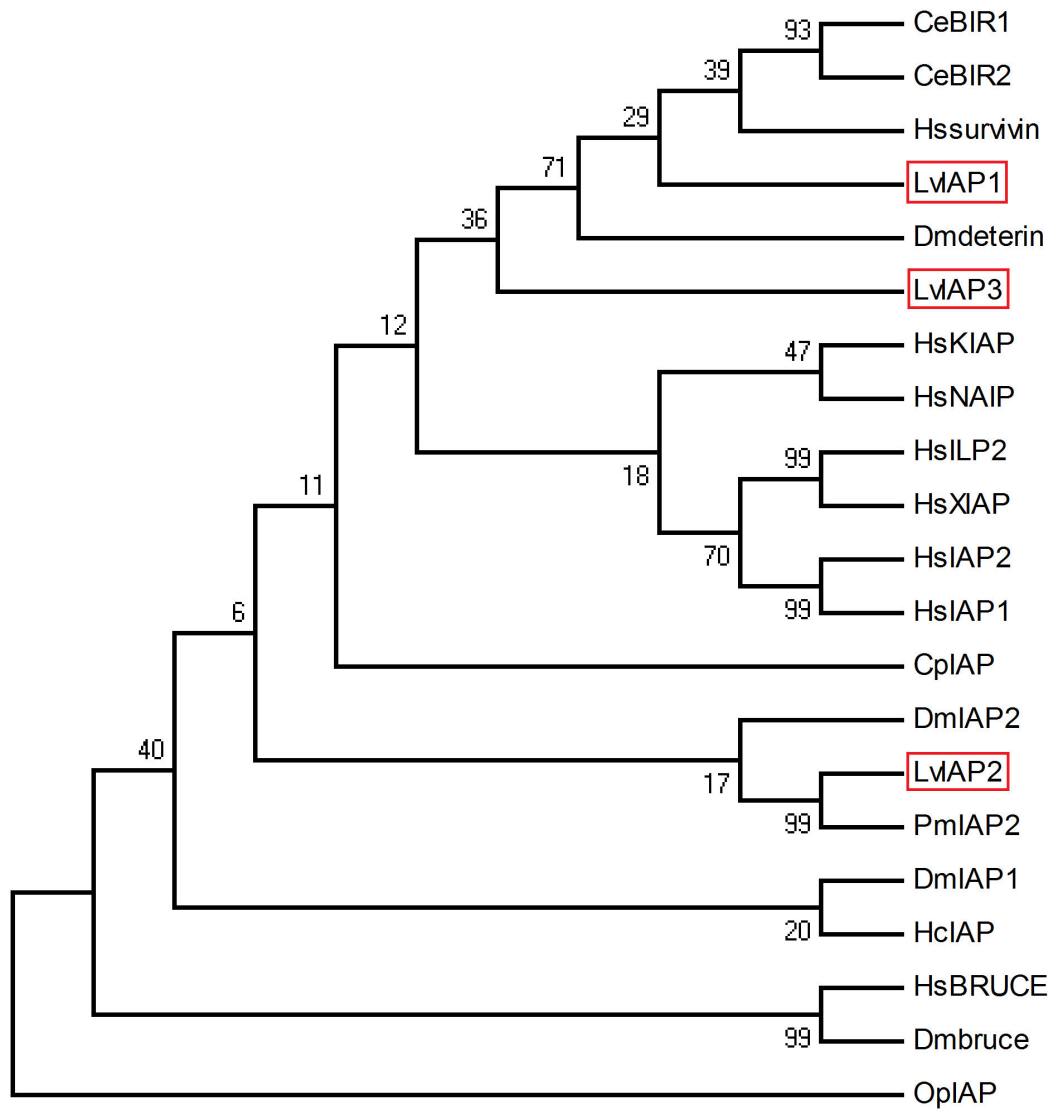
A phylogenetic tree of LvIAP1-3 with other IAPs. The numbers at the nodes indicate the bootstrap values. LvIAP1-3 are boxed with red lines. LvIAP1, *L* *. vannamei* IAP1 (Accession no. **AGC24178**); LvIAP2, *L* *. vannamei* IAP2 (Accession no. **AGC24179**); LvIAP3, *L* *. vannamei* IAP3 (Accession no. **AGC24180**); PmIAP2, *Penaeus* *monodon* IAP2 (Accession no. **ABO38431**); Hssurvivin, *Homo sapiens* survivin (Accession no. **NP_001125727**); HsILP2, *H. sapiens ILP2* (Accession no. **NP_203127**); HsKIAP, *H. sapiens* KIAP (Accession no. **NP_647478**); HsXIAP, *H. sapiens* XIAP (Accession no. **NP_001158**); HsIAP2, *H. sapiens* IAP2 (Accession no. **NP_001157**); HsIAP1, *H. sapiens* IAP1 (Accession no. **XP_003910644**); HsNAIP, *H. sapiens* NAIP (Accession no. **AAC62261**); HsBRUCE, *H. sapiens* BRUCE (Accession no. **XP_004029135**); DmIAP1, *Drosophila melanogaster* IAP1 (Accession no. **NP_524101**); DmIAP2, *D. melanogaster* IAP2 (Accession no. **Q24307**); Dmdeterin, *D. melanogaster* deterin (Accession no. **NP_650608**); Dmbruce, *D. melanogaster* bruce (Accession no. **NP_649995**); CeBIR1, *Caenorhabditis elegans* BIR1 (Accession no. **NP_506362**); CeBIR2, *C. elegans* BIR2 (Accession no. **NP_505949**); HcIAP, *Hyphantria* *cunea* nucleopolyhedrovirus IAP (Accession no. **YP_473308**); OpIAP, *Orgyia* *pseudotsugata* MNPV IAP (Accession no. **NP_046191**); CpIAP, *Cydiapomonella* granulovirus IAP (Accession no. **NP_148878**).

Using MEGA 4.0 software, we constructed NJ phylogenetic trees for IAPs from typical species. The NJ phylogenetic tree revealed two groups of shrimp IAPs: LvIAP1 and LvIAP3 were clustered with Dmdeterin and Hssurvivin in one group, and LvIAP2 clustered with DmIAP2 in another group (Figure 2).

### 3.3: Tissue distribution of LvIAP1-3 in healthy *L*. *vannamei*

**Figure 3:**
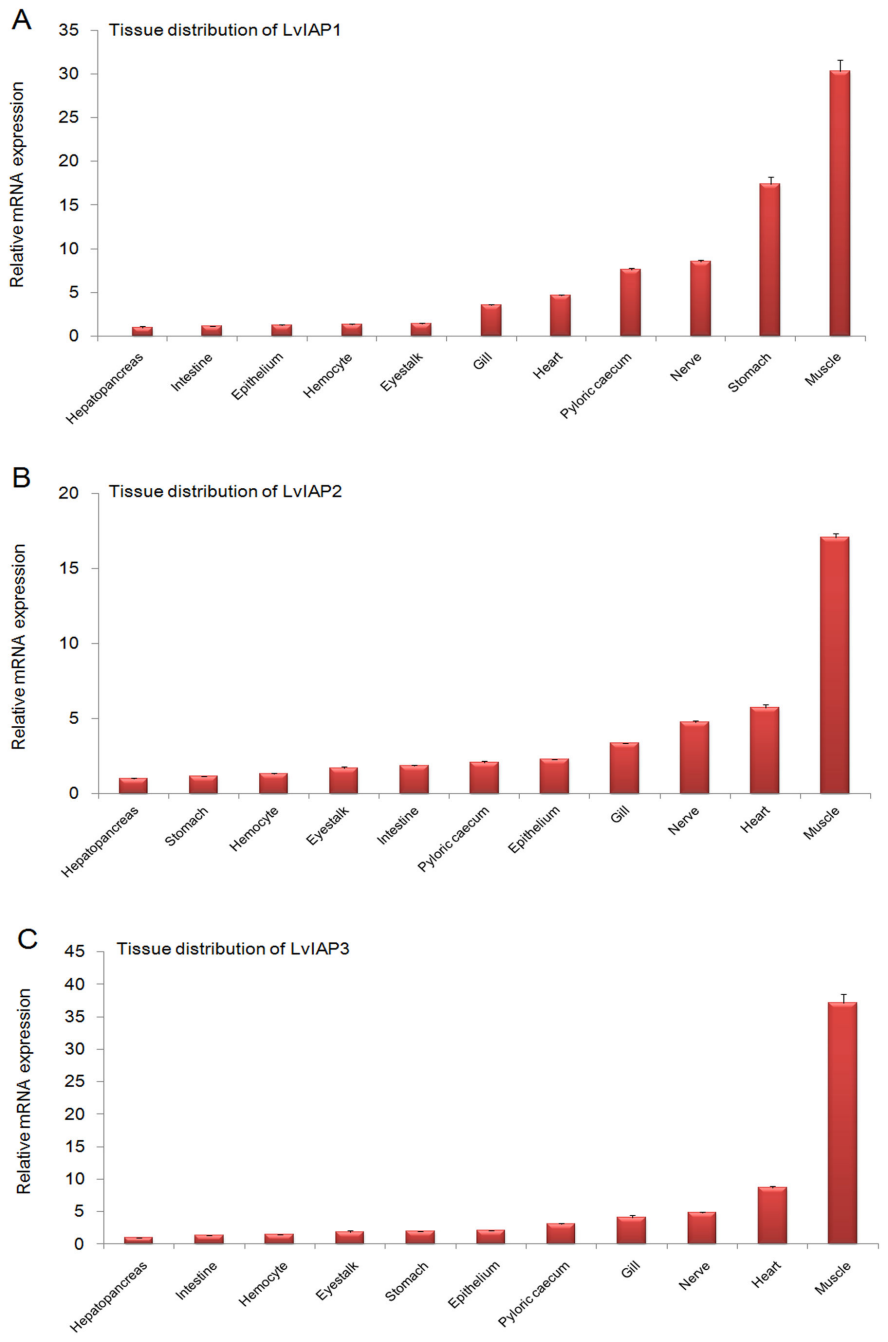
Tissue distribution of *LvIAP1* (A), *LvIAP2* (B), and *LvIAP3* (C) in healthy shrimps. The hemocytes, hepatopancreas, epithelium, intestine, eyestalk, stomach, gill, heart, pyloric cecum, nerve, and muscle were collected from healthy *L* *. vannamei* to extract total RNA for the tissue distribution analysis. The transcript expression levels of *LvIAP1-3* in the hepatopancreas were set to 1.0. The qPCR analysis was performed in triplicate for each sample. The data are expressed as the mean fold-changes (means ± S.E., n =3).

In healthy shrimp, when normalized to mRNA expression in the hepatopancreas (1.00-fold), *LvIAP1* was expressed at higher levels in the intestine (1.14-fold), epithelium (1.25-fold), hemocytes (1.34-fold), eyestalk (1.39-fold), gill (3.51-fold), heart (4.69-fold), pyloric cecum (7.64-fold), nerve (8.55-fold), stomach (17.42-fold), and muscle (30.34-fold) (Figure 3A), *LvIAP2* was expressed at higher levels in the stomach (1.14-fold), hemocytes (1.33-fold), eyestalk (1.70-fold), intestine (1.84-fold), pyloric cecum (2.09-fold), epithelium (2.27-fold), gill (3.34-fold), nerve (4.78-fold), heart (5.72-fold), and muscle (17.09-fold) (Figure 3B), *LvIAP3* was expressed at higher levels in the intestine (1.32-fold), hemocytes (1.50-fold), stomach (1.99-fold), eyestalk (1.91-fold), epithelium (2.07-fold), pyloric cecum (3.11-fold), gill (4.12-fold), nerve (4.84-fold), heart (8.71-fold), and muscle (37.15-fold) (Figure 3C). LvIAP1-3 mRNAs were expressed at significantly higher levels in the muscle but at lowest levels in the hepatopancreas.

### 3.4: Expression profiles of LvIAP1-3 after microbial challenge

**Figure 4:**
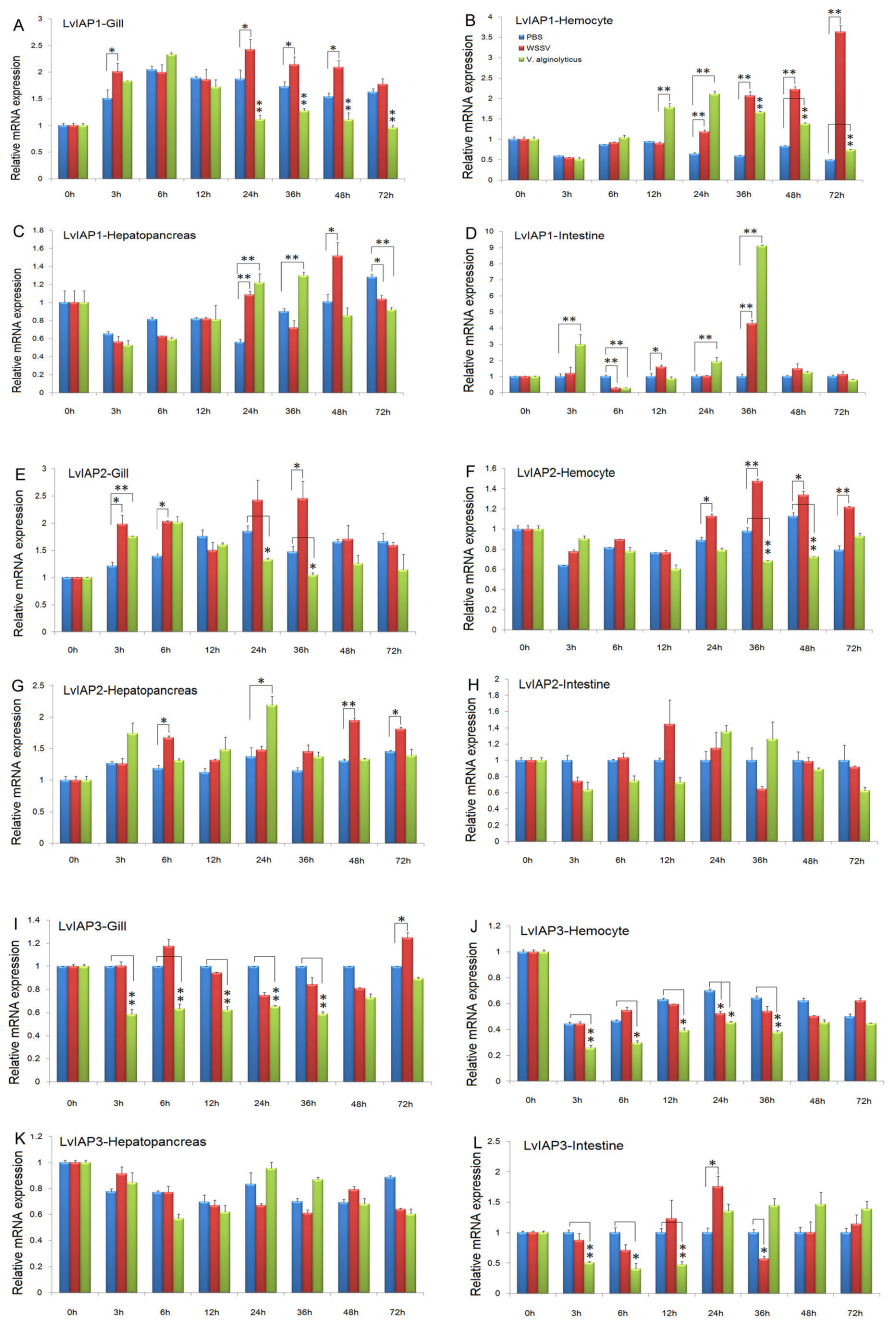
Temporal expression of *LvIAP1*, *LvIAP2*, and *LvIAP3* in the gill (A), hemocytes (B), hepatopancreas (C), and intestine (D) after PBS, WSSV, and *V* *. alginolyticus* infection. Healthy *L* *. vannamei* were injected intramuscularly at the third abdominal segment with PBS (control group), *V* *. alginolyticus* or WSSV inoculums. At different time points, five shrimp from each group were randomly selected, and the gill, hemocytes, hepatopancreas, and intestine were collected for qPCR analysis. The transcript expression levels of *LvIAP1-3* in the untreated control group (0 hpi) was set at 1.0 (*p < 0.05; **p < 0.01).

**Figure 5:**
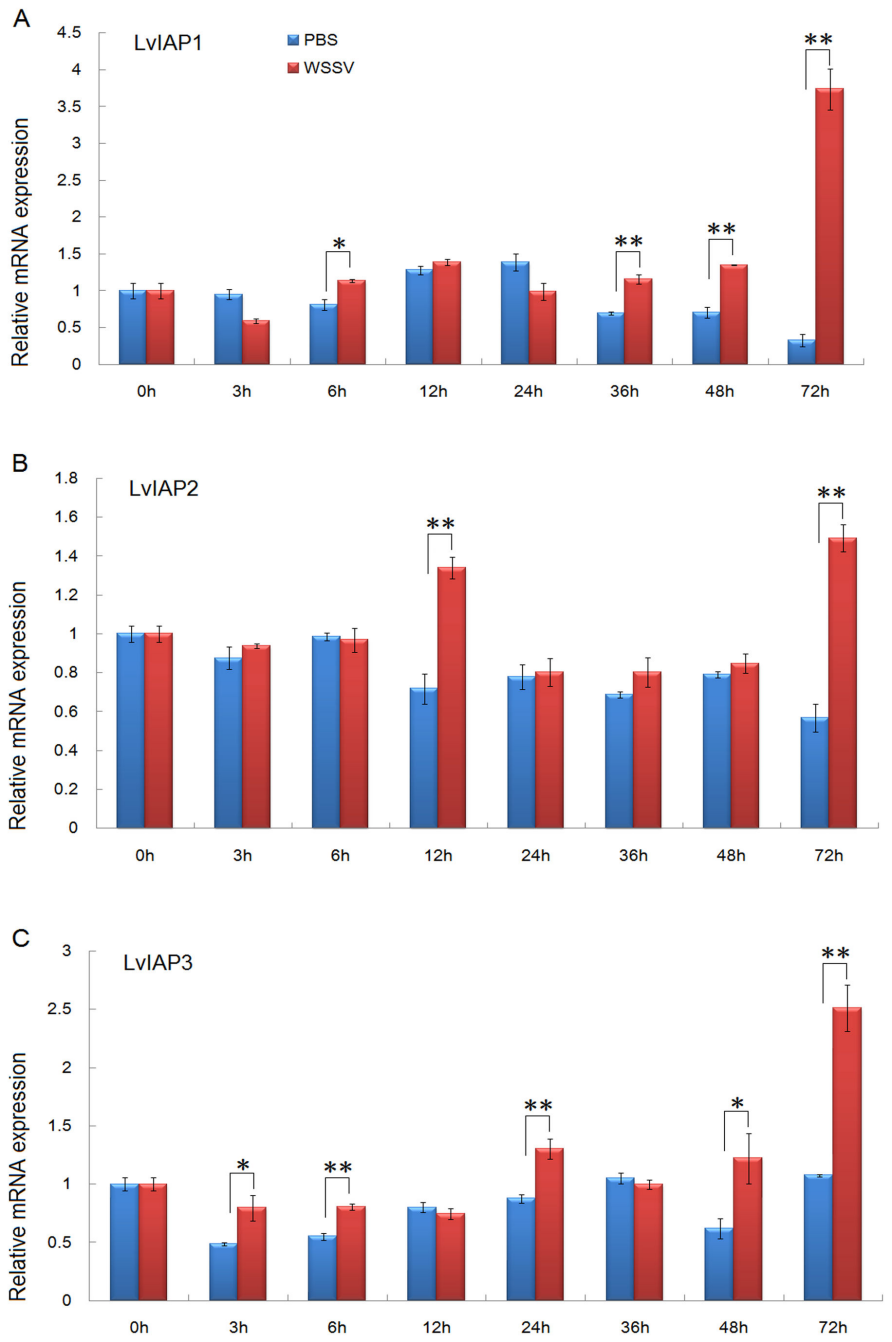
Temporal expression of *LvIAP1* (A)*, LvIAP2* (B), and *LvIAP3* (C) in the muscle after PBS and WSSV injection. *LvIAP1-3* expression in the untreated control group (0 hpi) was set at 1.0.

After WSSV infection, *LvIAP1* was upregulated in the gill, hemocytes, and intestine compared with the PBS-injected group (Figure 4A–D); *LvIAP2* was also upregulated in the gill, hemocytes, and hepatopancreas (Figure 4F–H); but LvIAP3 was only slightly upregulated in the gill and intestine (Figure 4I–L). LvIAP1-3 transcripts were significantly upregulated in the muscle after WSSV infection compared with the PBS-injected group (Figure 5). After *V* *. alginolyticus* infection, *LvIAP1* was downregulated in the gill but upregulated in the hemocyte, hepatopancreas, and intestine (Figure 4A–D); *LvIAP2* was downregulated in the gill and hemocyte (Figure 4E–H); and *LvIAP3* was downregulated in the gill, hemocyte, and intestine (Figure 4I–L).

### 3.5: Cellular localization of LvIAP1-3 in Drosophila S2 cells

**Figure 6:**
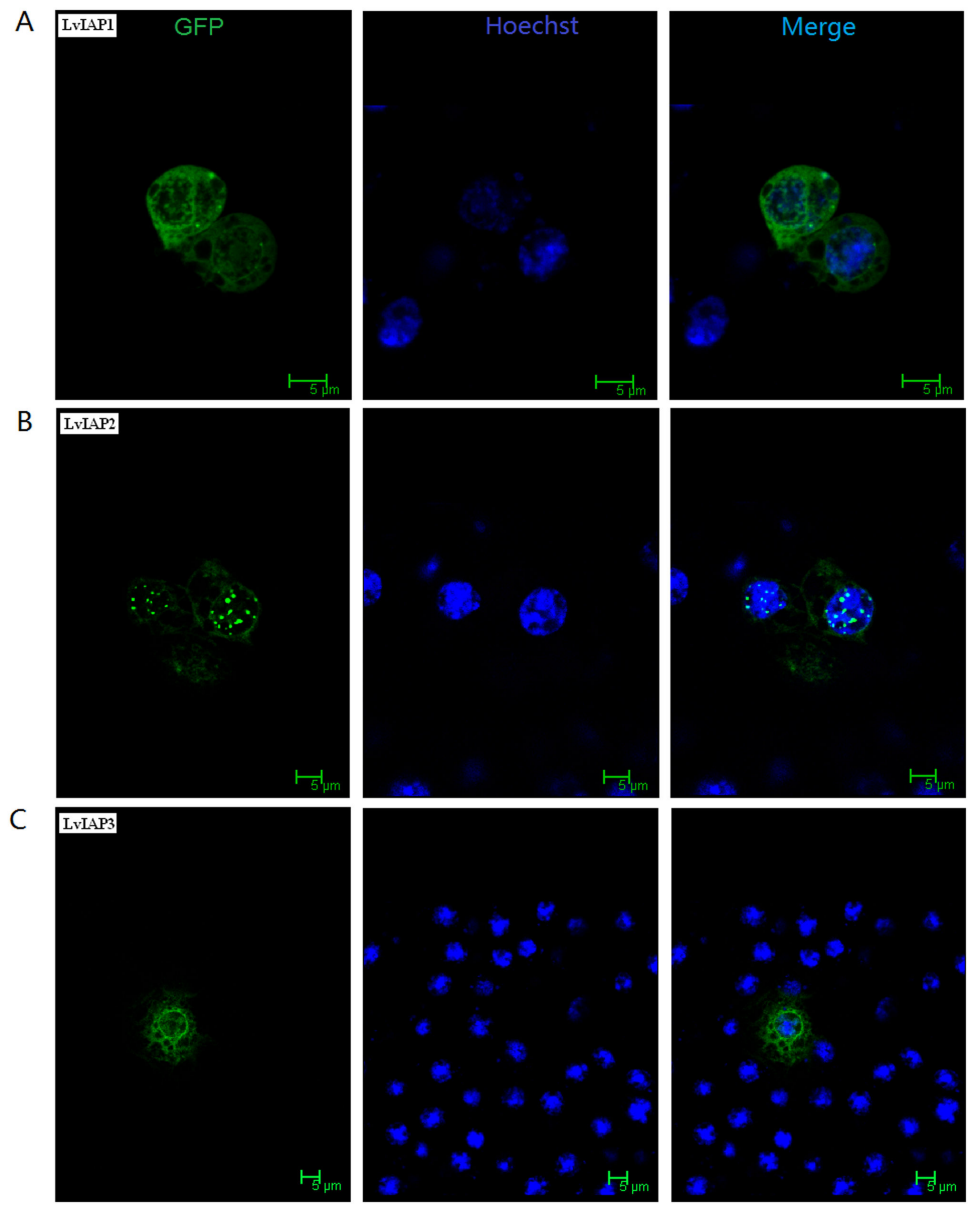
Subcellular localization of LvIAP1 (A), LvIAP2 (B), and LvIAP3 (C) in *Drosophila* S2 cells. *Drosophila* S2 cells were transfected with the pAc5.1-LvIAP1-3-GFP plasmids. At 36 hours post-transfection, the cover slips were washed, fixed, and stained with Hoechst 33258. The protein cellular localization was examined under a Leica laser scanning confocal microscope. The nuclei were visualized using the Hoechst stain (blue).

To examine the cellular localization of LvIAP1-3, LvIAP1, LvIAP2, and LvIAP3 were fused to GFP using a pAc5.1–N–GPF vector and expression of fusion proteins were observed using confocal microscopy. Both LvIAP1 and LvIAP3 fusion proteins were widely distributed in the cytoplasm and nucleus of *Drosophila* S2 cells, whereas LvIAP2 was distributed in the cytoplasm but appeared as speck-like aggregates in the nucleus (Figure 6).

### 3.6: The reduced expression of LvIAP1-3 in vivo by dsRNA-mediated gene silencing

**Figure 7:**
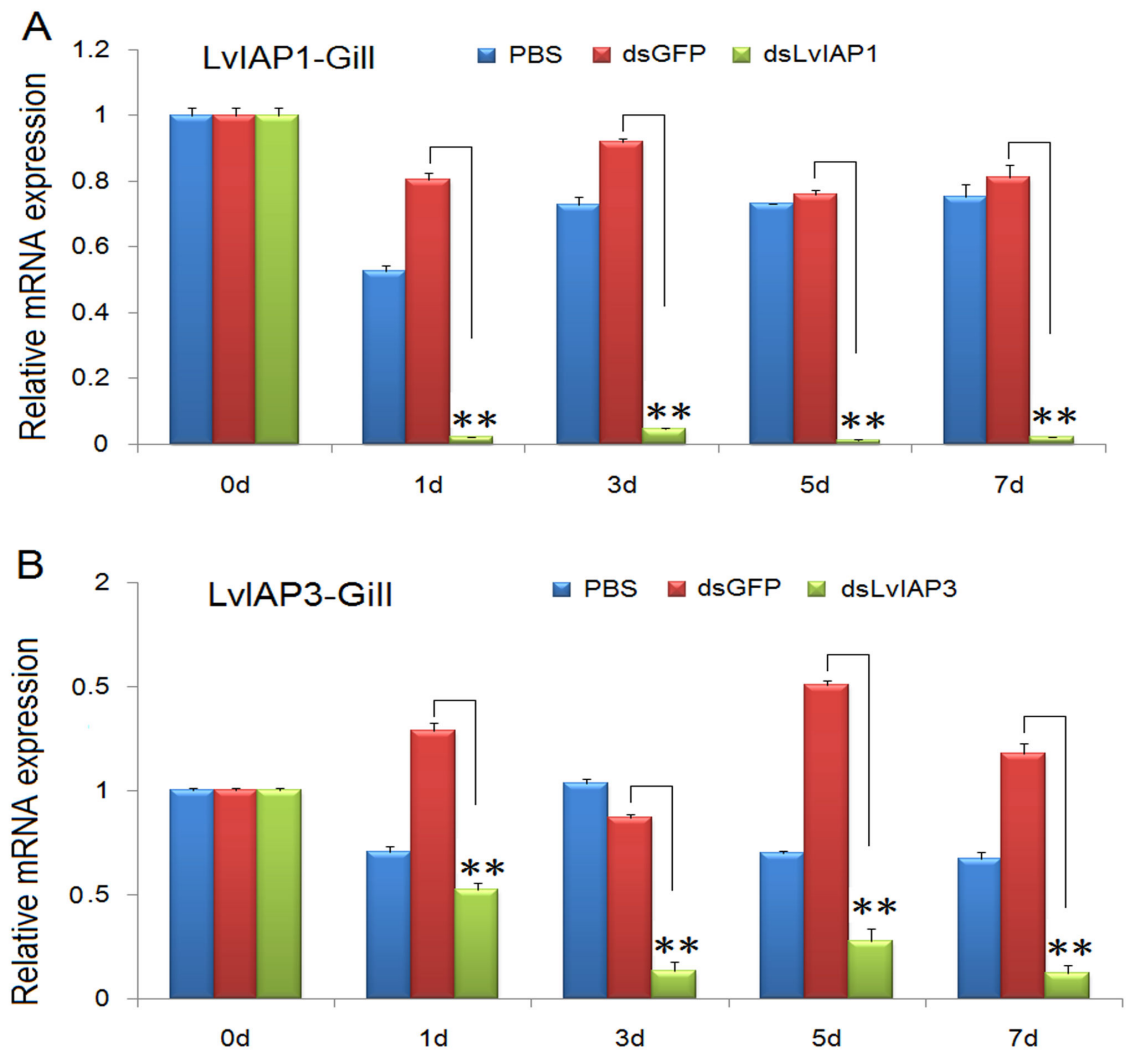
RNAi silencing of *LvIAP1* and *LvIAP3* in the gill of shrimp by dsRNAs. The experimental shrimp (1 g to 2 g) were intramuscularly injected with dsLvIAP1 or dsLvIAP3 (1 µg/g shrimp), whereas the control shrimp were injected with dsGFP and PBS separately. At the indicated time points after injection, total RNA was extracted from the gill and reverse transcribed to cDNA. The expression of *LvIAP1* and *LvIAP3* was determined using qPCR. The expression of *LvIAP1* and *LvIAP3* in the untreated control group (0 hpi) was set at 1.0.

**Figure 8:**
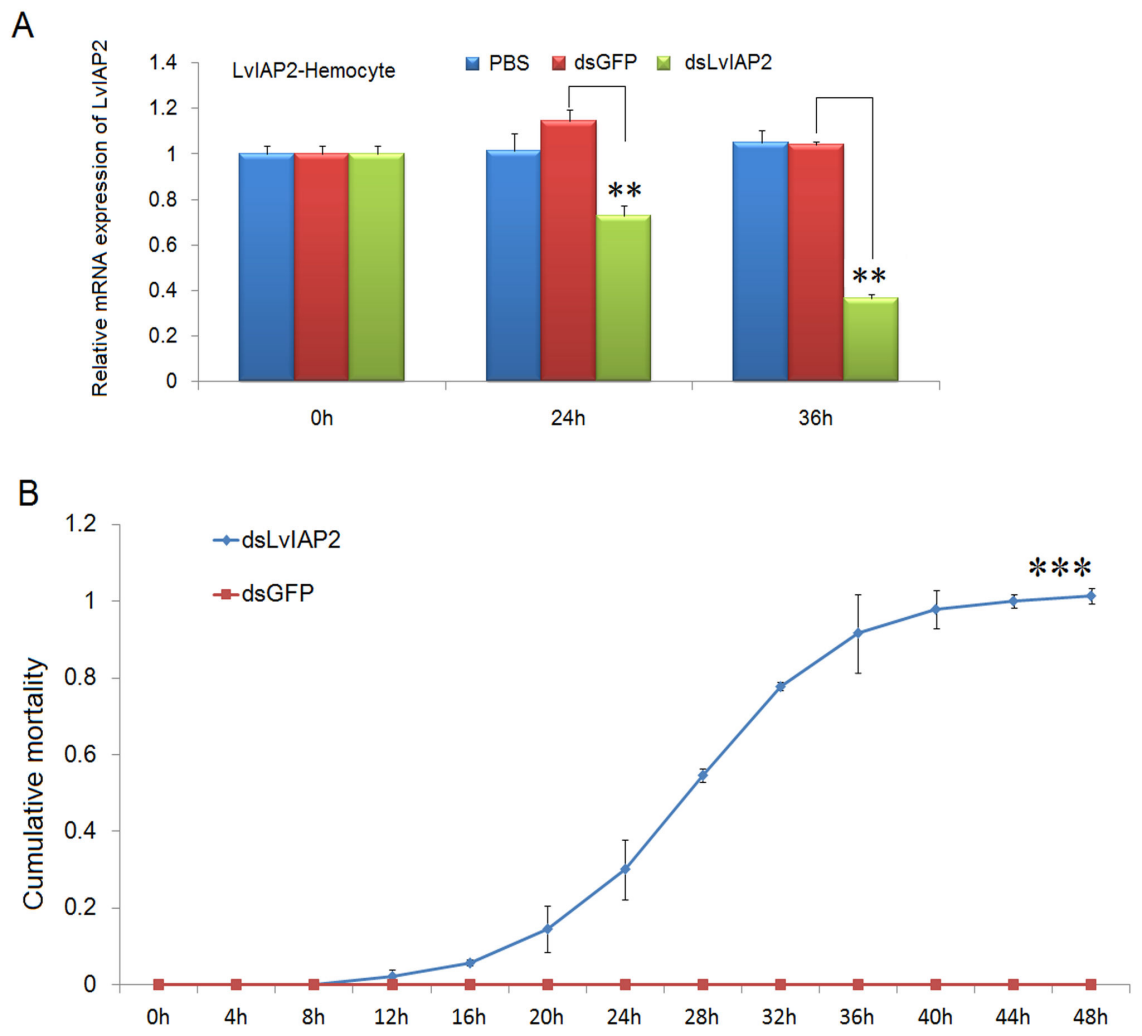
Silencing of LvIAP1 and LvIAP3 facilitates the reproduction of WSSV. At 48 hours after dsLvIAP1, dsLvIAP3, dsGFP or PBS injection, the shrimp were infected intramuscularly with a WSSV inoculum (107 copies/shrimp). At the indicated time points after WSSV infection, the gills of these shrimp were collected for qPCR analysis. The mRNA expression level of WSSV VP28 in the gills of shrimp injected with PBS, dsGFP (control), dsLvIAP1, or dsLvIAP3 after WSSV infection were determined using qPCR. The mRNA expression level of WSSV VP28 was normalized to that of *LvEF-1α* using the relative standard curve method for calculation of changes in gene expression as described in previous studies [43]..

To investigate the function of *LvIAP1-3* in shrimp defense against WSSV infection, dsRNA-mediated gene silencing experiments were performed. dsLvIAP1-3 (1 µg/g shrimp) were intramuscularly injected into shrimp separately, in the experimental groups, while injection of dsGFP or PBS was used in the control groups. In the gill, the expression of *LvIAP1* and *LvIAP3* was significantly suppressed at 24, 72, 120 and 144 hpi (Figure 7), while the expression of *LvIAP2* was silenced in hemocytes, but not in the gill (Figure 8A). Intriguingly, the *LvIAP2*-silenced shrimp died within 48 hours after dsLvIAP2 injection (Figure 8B).

### 3.7: WSSV VP28 expression in dsRNA-injected *L*. *vannamei*

**Figure 9:**
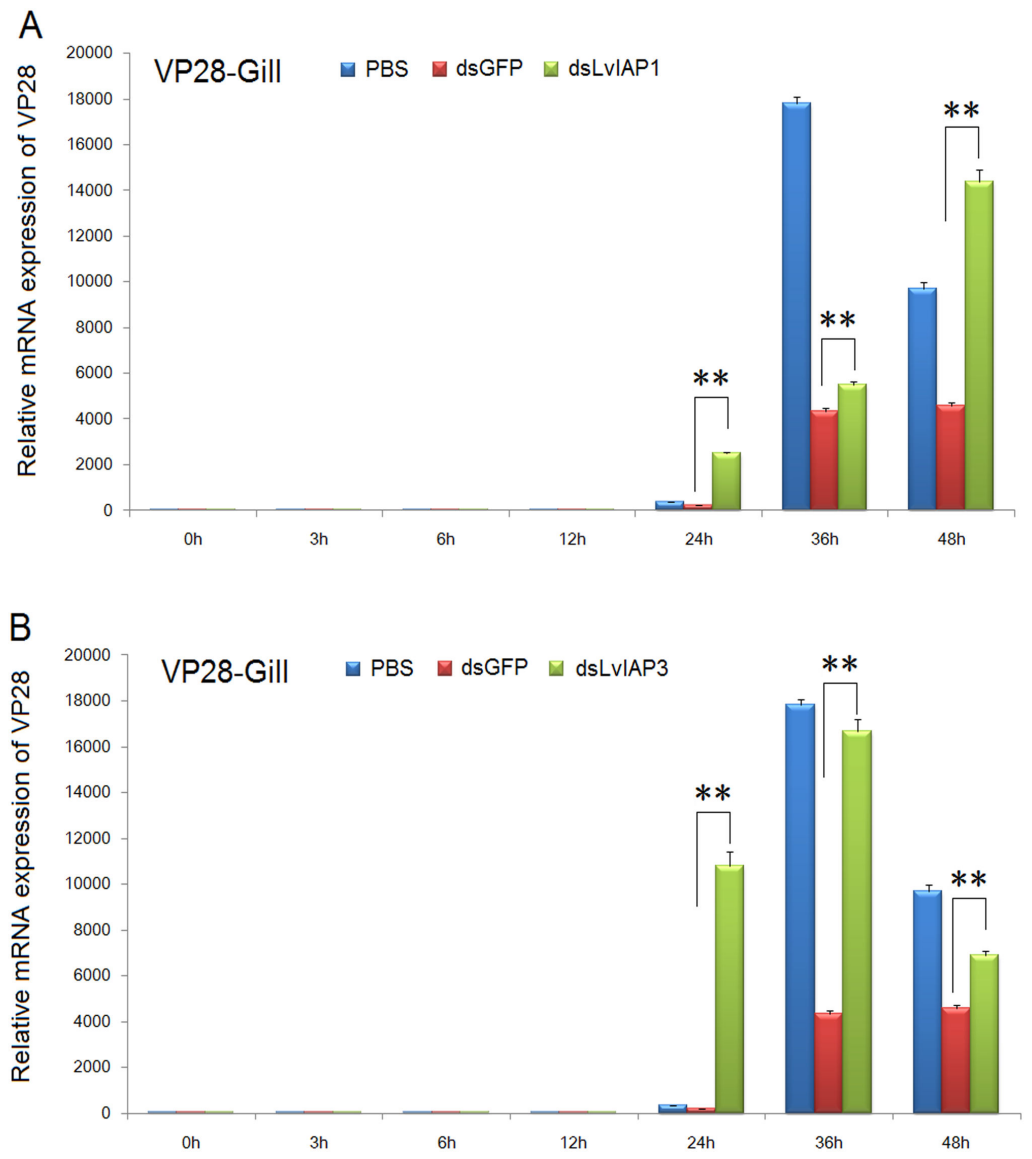
Silencing of *LvIAP2* led to 100% mortality in *L. vannamei* within 48 hours. (A) Expression of *LvIAP2* in the hemocyte of dsLvIAP2-injected shrimp was significantly suppressed by dsRNA-mediated RNAi. (B) The cumulative mortality rate of shrimp injected with dsGFP (control) or dsLvIAP2. The chi-square statistic was calculated to assess the differences in mortality rates by comparing the mortality of dsLvIAP2- injection group with that of the dsGFP- injection group (***p < 0.001).

To further evaluate the role of LvIAP1 and LvIAP3 in shrimp defense against WSSV infection, we performed WSSV infection experiments in dsRNA-injected *L* *. vannamei* . At 48 hours after dsRNA injection, *L* *. vannamei* were infected with WSSV. We observed that at 24, 36 and 48 hpi, the expression of WSSV *VP28* in the gill of the dsLvIAP1- and dsLvIAP3-injected groups was dramatically higher than that in the dsGFP- or PBS-injected group (Figure 9). At 24 hpi, the expression of *VP28* was low in the PBS and dsGFP-injected groups, but in the dsLvIAP1- and dsLvIAP3-injected groups, the expression of *VP28* was high, suggesting that the silencing of *LvIAP1* and *LvIAP3* accelerates WSSV infection (Figure 9). We also observed that at 24 and 36 hpi, the expression of WSSV *VP28* was higher in *LvIAP1*-silenced shrimp that in LvIAP3 silenced shrimp (Figure 9). In *LvIAP1*-silenced shrimp, WSSV *VP28* was gradually increased, but in *LvIAP3*-silenced shrimp, the expression of WSSV *VP28* diminished (Figure 9). These results suggest that LvIAP1 and LvIAP3 are involved in the host defense against WSSV infection differently.

### 3.8: The activation of the promoters of NF-κB pathway-controlled AMPs by LvIAP2 in Drosophila S2 cells

**Figure 10:**
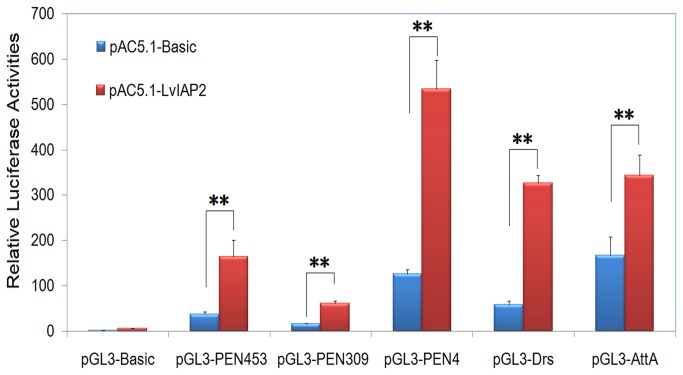
Activation of the promoters of *Drosophila* and shrimp AMP genes by overexpression of LvIAP2 in *Drosophila* S2 cells. *Drosophila* S2 cells were transfected with a protein expression vector (pAC5.1 empty vector or pAC5.1-LvIAP2 vector), a luciferase reporter plasmid (pGL3-Basic, pGL3-PEN453, pGL3-PEN309, pGL3-PEN4, pGL3-Drs, or pGL3-AttA), and pRL-TK *Renilla* luciferase plasmid (as an internal control) (Promega, USA). Thirty-six hours later, the cells were harvested for examination of luciferase activities using the dual luciferase reporter assay system (Promega, USA). All data are representative of three independent experiments. The bars indicate the mean ± S.D. of the luciferase activity (n = 3).

A comparison with four *Drosophila* IAPs (DIAPs) revealed that LvIAP2 is similar to DIAP1 and DIAP2 in domain structure and protein sequence (Figure 1D). DIAP1 plays an essential role in regulation of apoptosis, and DIAP2 is required for the IMD pathway in AMP regulation but is dispensable for *Drosophila* survival [16,53]. A previous study indicated that a shrimp IAP homolog of LvIAP2 evaluated in this study is essential for shrimp survival [42]. In this study, we investigated whether LvIAP2 functions in AMP regulation through the IMD-mediated NF-κB pathway in *Drosophila* S2 cells. The results of the dual luciferase reporter assays indicated that overexpression of LvIAP2 significantly induced the promoter activities of *Drosophila* AMPs, including *Drosomycin* (*Drs*) (5.57-fold) and *Attacin A* (*AttA*) (2.06-fold), *P* *. monodon* AMP *Penaeidin* (*PEN309* and *PEN453* at 3.76 and 4.47-fold, respectively), and *L* *. vannamei* AMP *Penaeidin4* (*PEN4*) (4.22-fold) (Figure 10). However, overexpression of neither LvIAP1 nor LvIAP3 affected the activities of these AMP promoters (result not shown).

### 3.9: The activation of the promoters of WSSV069, WSSV303, and WSSV371 by LvIAP2 in Drosophila S2 cells

**Figure 11:**
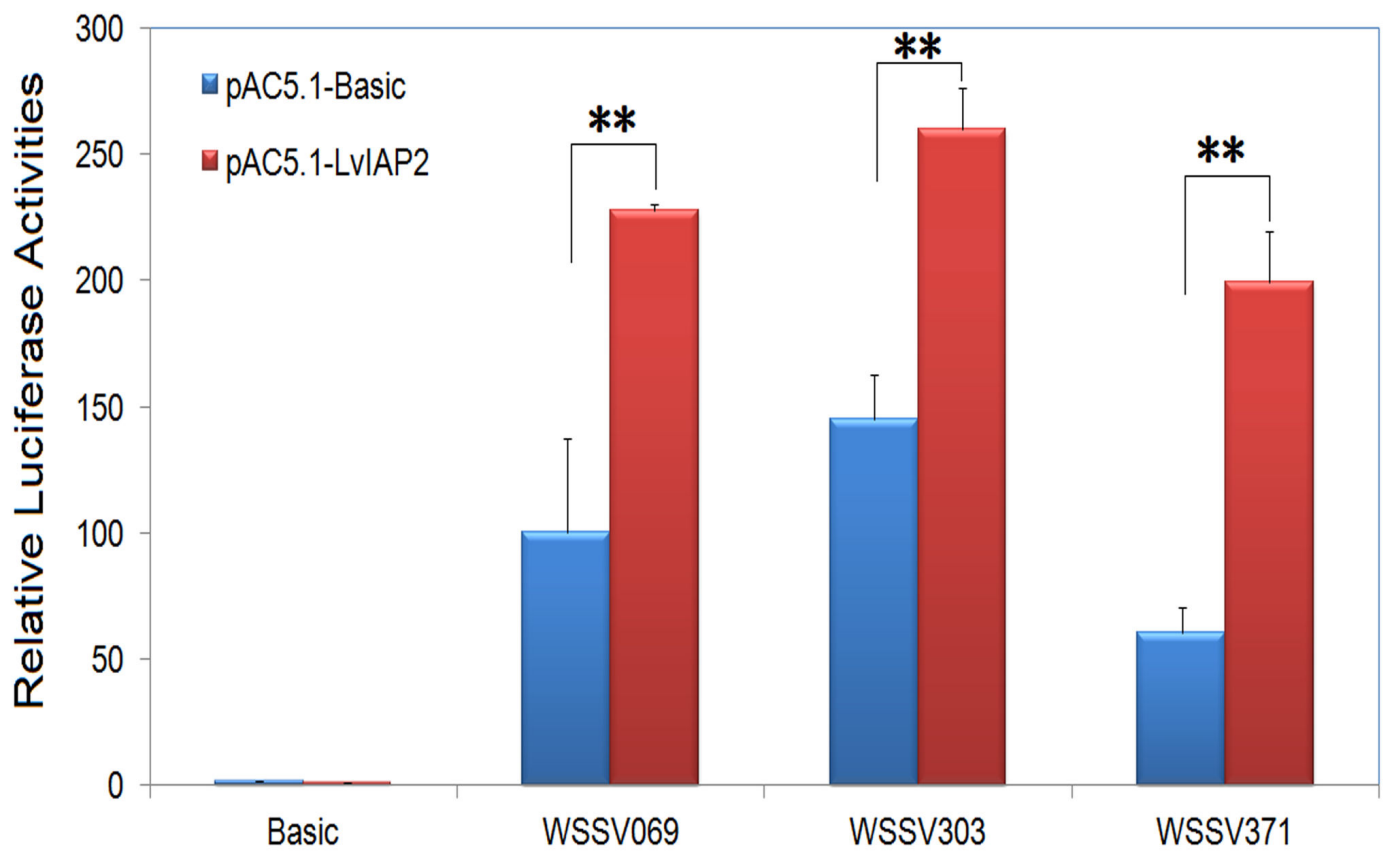
Activation of the promoters of *WSSV069* (*ie1*), *WSSV303*, and *WSSV371* by overexpression of LvIAP2 in *Drosophila* S2 cells.

In a previous study, we showed that several viral genes, including WSSV069, WSSV303, and WSSV371 that possess NF-κB binding sites in the promoter regions, were regulated through the NF-κB signaling pathway [48]. Here, we also showed that overexpression of LvIAP2 in *Drosophila* S2 cells activatedthe promoters of *WSSV069* (*ie1*), *WSSV303*, and *WSSV371* by 2.27-, 1.79-, and 3.30-fold, respectively (Figure 11).

### 3.10: The expression of LvPEN2-4, Lvlysozyme, Lvcrustin1-3, LvVICP1-2, and LvALF1-3 in LvIAP2-silenced shrimp

**Figure 12:**
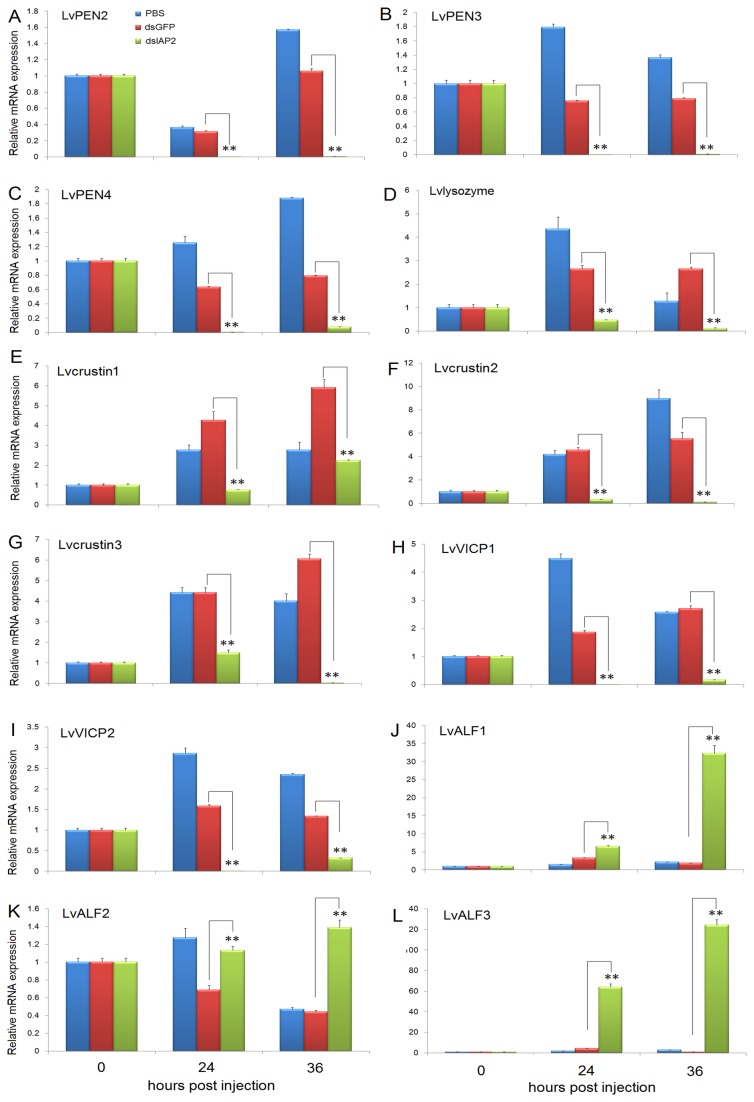
Silencing of *LvIAP2* led to decrease in expression of *LvPEN2*, *LvPEN3*, *LvPEN4*, *Lvlysozyme*, *Lvcrustin1*, *Lvcrustin2*, *Lvcrustin3*, *LvVICP1*, and *LvVICP2* but increase in expression of *LvALF1*, *LvALF2*, and *LvALF3* in the hemocytes. Shrimps were injected with PBS, dsGFP (control), or dsLvIAP2, and the hemocytes were collected at the indicated time points for total RNA isolation and first-stranded cDNA preparation. The expression levels of *LvPEN2*, *LvPEN3*, *LvPEN4*, *Lvlysozyme*, *Lvcrustin1*, *Lvcrustin2*, *Lvcrustin3*, *LvVICP1*, *LvVICP2*, *LvALF1*, *LvALF2*, and *LvALF3* were determined by qPCR.

To further confirm LvIAP2’s function in shrimp AMP regulation, we examined the expression of shrimp AMPs, including *PENs*, *lysozyme*, *crustins*, *VICPs*, and *ALFs*, in *LvIAP2*-silenced shrimp. We observed that in the hemocytes of *LvIAP2*-silenced shrimp, the expression of *LvPEN2-4*, *Lvlysozyme*, *Lvcrustin1-3*, and *LvVICP1-2* was significantly reduced compared with that of dsGFP-injected shrimp at 24 and 36 hpi(Figure 12A–I). However, in the hemocytes of *LvIAP2*-silenced shrimp, the expression of *LvALF1* was upregulated 1.97- and 17.08-fold at 24 and 36 hpi, respectively (Figure 12J); the expression of *LvALF2* was upregulated 1.65- and 3.13-fold at 24 and 36 hpi, respectively (Figure 12K); the expression of *LvALF3* was upregulated 15.00- and 123.87-fold at 24 and 36 hpi, respectively (Figure 12L).

## Discussion

Apoptosis is a tightly regulated process in which excess or damaged cells are eliminated to maintain tissue homeostasis [1,3,6,21]. Apoptosis is also a major defense mechanism to remove unwanted and potentially dangerous cells, such as virus-infected cells [3,5,54,55]. Shrimps use apoptosis in defense against WSSV infection, and WSSV encodes two anti-apoptosis proteins, AAP-1 (ORF390 or WSSV449) and WSSV222, to subvert host apoptosis responses to facilitate viral replication [26,27,29,39]. Inhibitors of apoptosis proteins (IAPs) inhibit the activity of caspases, the primary executor of the apoptosis program, and play important roles in regulating the progression of apoptosis from insects to humans [1,6,56]. In addition to apoptosis regulation, IAPs also participate in diverse cellular activities, such as signal transduction, innate immunity, and mitosis [21,57,58]. In this study, three IAPs from *L* *. vannamei* (LvIAP1-3) were cloned and characterized. Using dsRNA-mediated gene silencing, we investigated the functions of IAPs in WSSV infection and shrimp AMP regulation.

*LvIAP1-3* mRNAs were primarily expressed in the muscle and upregulated after WSSV infection (Figures 3-5). After WSSV infection, *LvIAP1* was upregulated in the gill, hepatopancreas, hemocytes, and intestine (Figure 4); *LvIAP2* was upregulated in the gill, hepatopancreas, hemocytes, but not in the intestine (Figure 4); *LvIAP3* did not show significant changes (Figure 4). In *LvIAP1*- or *LvIAP3*-silenced shrimp, the expression of WSSV *VP28* increased dramatically compared with that in the dsGFP control group (Figure 9), suggesting protective roles of LvIAP1 and LvIAP3 in shrimp defense against WSSV infection. In the present study, dsLvIAP1 and dsLvIAP3 were injected followed by WSSV infection. The silencing of *LvIAP1* and *LvIAP3* in the early stages during WSSV infection might promote apoptosis to facilitate the spread of virus progeny to neighboring cells. However, the detailed mechanism underlying this process needs further investigation (e.g., silencing LvIAP1 and LvIAP3 at different stages during WSSV infection and observing the replication of WSSV). Interestingly, LvIAP2 was only effectively silenced in the hemocytes, but not in the gill, and the *LvIAP2*-silenced shrimp died within 48 hours after dsLvIAP2 injection (Figure 8). This phenomenon was also observed in a recently published paper, in which the authors concluded that reduction in the number of hemocytes in *IAP2*-silenced shrimp reflects extensive apoptosis [42]. We also observed that the circulating hemocytes were dramatically reduced in *LvIAP2*-silenced shrimp (results not shown). Thus, hemocytes play a pivotal role in shrimp survival, and LvIAP2 plays a central role in regulation of shrimp hemocyte apoptosis. LvIAPs were also cloned in a recent report [42] and the function of LvIAP2 in shrimp haemocyte apoptosis is well studied. In this study, we investigated induced expression of LvIAP1-3 by WSSV infection and their potential involvements in host defense against viral infection. Particularly, we further studied the function of LvIAP2 in regulation of shrimp AMPs.

A comparison with four *Drosophila* IAPs (DIAPs) revealed that LvIAP2 is structurally related to DIAP1 and DIAP2 and is most similar to DIAP2 (Figure 1). DIAP1 is essential for *Drosophila* cell survival *in vivo* and *in vitro*, whereas DIAP2 is required for the IMD pathway in AMP regulation, but is dispensable for *Drosophila* survival [16,53]. In the present study, we observed that, similar to DIAP2, LvIAP2 activated the IMD pathway through the induction of the promoter activities of *Drosophila* and shrimp AMPs in *Drosophila* S2 cells (Figure 10). In addition, shrimp AMPs, such as *PENs*, *lysozyme*, *crustins*, *VICPs*, and *ALFs*, were significantly downregulated in the hemocytes of *LvIAP2*-silenced shrimp (Figure 12). The increase expression of ALFs might reflect different regulation mechanisms of various AMPs, which has also been recently observed in other studies [59]. The silencing of *Cactus* (a shrimp IκB homolog and potential negative regulator of the shrimp Toll pathway) in Chinese shrimp *Fenneropenaeus* *chinensis* downregulated *ALF* expression, consistent with the upregulation of *ALF* expression in the hemocytes of *LvIAP2*-silenced *L* *. vannamei* [59]. The silencing of *LvTollip*, a potential negative regulator of the shrimp Toll pathway, also downregulates the expression of another shrimp AMP, *PEN2* [60]. These results suggest that shrimp AMPs are regulated through the Toll/IMD-NF-κB signaling pathway, but with different mechanisms. We propose that *ALFs* might have different regulation mechanisms from those of *PENs*, *lysozyme*, *crustins*, and *VICPs*.

In the present study, we cloned two new members of the recently identified shrimp AMP *VICPs* ( *Vibrio* *penaeicidae* -*induced cysteine and proline-rich peptide*, called *Stylicins* in Paciﬁc blue shrimp *Litopenaeusstylirostris* ) [61]. *Litopenaeusstylirostris* Stylicin displays strong antifungal activity against *Fusarium oxysporum*, a pathogenic fungus of shrimp. The regulation of *LvVICPs* through LvIAP2 might suggest that the shrimp IMD pathway is involved in antifungal responses. In *Drosophila* , the Toll pathway, but not the IMD pathway, primarily regulates antifungal and anti-Gram-positive bacterial responses [10,11]. Thus, the antibacterial and antifungal mechanisms might be different in the Toll and IMD pathways in *Drosophila* and shrimps. Notably, the expression of all the *ALFs*, including *LvALF1*, *LvALF2*, and *LvALF3*, was upregulated in the hemocytes of *LvIAP2-*silenced shrimp. Thus, LvIAP2 might negatively regulate the expression of *ALFs*.

Although LvIAP1-3 possess the characteristic baculoviral IAP repeat (BIR) domain, these proteins also differed in many aspects. LvIAP1 has one BIR domain, similar to mammalian survivin and *Drosophila* deterin, whereas LvIAP2 contains three BIR domains and a C-terminal RING domain. Therefore, LvIAP2 is structurally similar to mammalian XIAP, cIAP1, and cIAP2 and *Drosophila* IAP2 (Figure 1D), which possess three BIR domains and a RING domain and are involved in NF-κB activation [7,8,21–24]. LvIAP3 is a completely novel member of the IAP family proteins, with two BIR domains, which is not similar to any known mammalian or insect IAPs. Our results suggest that LvIAP2 possesses dual functions in both the DIAP1-mediated apoptosis and DIAP2-mediated NF-κB activation of the IMD pathway [41,42]. Considering the protein identity and domain structure of LvIAP2 and biological function in AMP regulation by LvIAP2, we propose that LvIAP2 is the homolog of DIAP2, but not DIAP1. Therefore, we refer to this molecule as LvIAP2 in the present study.

Apoptosis-related genes, such as Pmcaspase, have been targets of small molecule drugs to improve the apoptotic activity of shrimp hemocytes for the inhibition of WSSV infection [27,62]. Thus, in future studies, the detailed functions of shrimp IAPs in different stages during WSSV infection should be investigated to provide information for the development of drugs targeting shrimp IAPs to manipulate apoptosis as novel strategies for the prevention and control of WSSV infection.

## Supporting Information

Figure S1 **The promoter sequences (shaded regions) of *Drosophila* Attacin *A* (AttA), Drosomycin (Drs), *Litopenaeusvannamei* *Penaeidin4*, and *Penaeus* *monodon* *penaeidin* (two types, PmPEN309 and PmPEN453) were shown.** The primers used in luciferase reporter construction were also provided. Protocols for dual luciferase reporter assays are as followings:
1) *Drosophila* S2 cells were maintained at 28°C in standard Drosophila medium (Serum-Free Medium; Invitrogen, USA), supplemented with 10% fetal bovine serum (FBS) and 1% Penicillin–Streptomycin solution.
2) Twenty-four hours prior to transfection, the cells were seeded in a 24-well culture plate in 2ml medium at 1×10^6^ cells/ml.
3) Transfections were conducted using Effectene Transfection Reagent (Qiagen, Germany) following the protocols. The protein expression vector (pAc5.1-LvIAP2) was co-transfected with pGL3 luciferase vectors (pGL3-AttA, pGL3-Drs, pGL3-LvPEN4, pGL3-PmPEN453, pGL3-PmPEN309, pGL3-WSSV069, pGL3-WSSV303, or pGL3-WSSV371) to study the activation of the reporters by LvIAP2. The pRL-TK *Renilla* luciferase vector was used as an internal control.
4) *Drosophila* S2 cells were harvested and lysed 36 hours after transfection for examination of dual luciferase activities using the dual luciferase reporter assay system (Promega, USA).
(DOCX)

Figure S2 **cDNA sequences of WSSV VP28 and *Litopenaeusvannamei* AMPs including *LvPEN2-4*, *Lvlysozyme*, *Lvcrustin1-3*, *LvALF1-3*, and *LvVICP1-2*.** The qPCR primers are also provided and underlined in the cDNA sequences. The ORFs of AMPs were shaded.
(DOCX)
