# Supplementary material for: Analysis of Expression, Cellular Localization, and Function of Three Inhibitors of Apoptosis (IAPs) from Litopenaeus vannamei during WSSV Infection and in Regulation of Antimicrobial Peptide Genes (AMPs)
Source: PLoS One. 2013 Aug 14;8(8):e72592. doi: 10.1371/journal.pone.0072592 (PMC3743791; doi:10.1371/journal.pone.0072592)
Supplement: Figure S1 — The promoter sequences (shaded regions) of Drosophila Attacin A (AttA), Drosomycin (Drs), Litopenaeusvannamei Penaeidin4, and Penaeus monodon penaeidin (two types, PmPEN309 and PmPEN453) were shown. The primers used in luciferase reporter construction were also provided. Protocols for dual luciferase reporter assays are as followings: 1) Drosophila S2 cells were maintained at 28°C in standard Drosophila medium (Serum-Free Medium; Invitrogen, USA), supplemented with 10% fetal bovine serum (FBS) and 1% Penicillin–Streptomycin solution. 2) Twenty-four hours prior to transfection, the cells were seeded in a 24-well culture plate in 2ml medium at 1×10^6^ cells/ml. 3) Transfections were conducted using Effectene Transfection Reagent (Qiagen, Germany) following the protocols. The protein expression vector (pAc5.1-LvIAP2) was co-transfected with pGL3 luciferase vectors (pGL3-AttA, pGL3-Drs, pGL3-LvPEN4, pGL3-PmPEN453, pGL3-PmPEN309, pGL3-WSSV069, pGL3-WSSV303, or pGL3-WSSV371) to study the activation of the reporters by LvIAP2. The pRL-TK Renilla luciferase vector was used as an internal control. 4) Drosophila S2 cells were harvested and lysed 36 hours after transfection for examination of dual luciferase activities using the dual luciferase reporter assay system (Promega, USA). (DOCX) [file pone.0072592.s001.docx]

**Supplementary data**

Fig.S1. The promoter sequences (shaded regions) of *Drosophila* *Attacin A (AttA)*, *Drosomycin* (Drs), *Litopenaeus vannamei* *Penaeidin4,* and *Penaeus monodon penaeidin* (two typles, PmPEN309 and PmPEN453) were shown as followings. The primers used in luciferase reporter construction were also provided. Protocols for dual luciferase reporter assays are as followings:

1) *Drosophila* S2 cells were maintained at 28°C in standard Drosophila medium (Serum-Free Medium; Invitrogen, USA), supplemented with 10% fetal bovine serum (FBS) and 1% Penicillin–Streptomycin solution.

2) Twenty-four hours prior to transfection, the cells were seeded in a 24-well culture plate in 2ml medium at 1×10^6^ cells/ml.

3) Transfections were conducted using Effectene Transfection Reagent (Qiagen, Germany) following the protocols. The protein expression vector (pAc5.1-LvIAP2) was co-transfected with pGL3 luciferase vectors (pGL3-AttA, pGL3-Drs, pGL3-LvPEN4, pGL3-PmPEN453, pGL3-PmPEN309, pGL3-WSSV069, pGL3-WSSV303, or pGL3-WSSV371) to study the activation of the reporters by LvIAP2. pRL-TK were used as internal control.

4) *Drosophila* S2 cells were harvested and lysed 36 hours after transfection for examination of dual luciferase activities using the dual luciferase reporter assay system (Promega, USA).

>*Drosophila* AttA Promoter

GCCATCAGGCCACCACCCATTCTGCCCGCCTAAAGATGTGTGCATACCGCGGAGAAGTCATCCGATCAAATTTGTTTTGAAAAATCTTTATAAAAATTGTGAATTTTTTACTTTCTGCAAACAGTAAGCAATAAACACACGAAAGACAGCAATTAATAATCTTCAATCAATTGTGACACAATGAGGGGTTCCCATCGCTTATCAGCGGTTTTTGTACCGAATCTGCTGAGCTCTAGAGCTGATAAGAAATATACTTGCTCAAAACAAAACCACAAAAGTCACGTTTGAGAGAAAAAAAGCCTAAACGAATTTAATTCGCGACTCATATGAATCACAAACCTGTTCTATAGCACGTTCTTCTTAAATTCTAGCGAAATAACCAGATGGCTGCAAATCATAATGAATGGGTTTGTCCCCTAAAAAAAACGAACTGACAAGCCCCCTTATAAAACTTATTTATTAATAGATTAGTTCGTATATAATTGCATATGTAAATACTTTAAATAGAACAAATTATTACGACATTTAAAAATATATATCCTGGTTTTTAAAAACAGGGTTTGAAAAAAATGTTTATAAGCTAATTACCTGGTAGATTATATTTTTCAGTGCAAACTTTTCGTTGACACTGCGGGTTAAAGTTTCCGCTCTCCTCTCTTTCGGCTCGCATCCTTTTTGCCCGCTCCTGCGCAGAAAATTCAATTAGGATTCGGCTGAAACTTCACTCAAATCCTGCCGCTCTCACCGTCGCTCTTTATTTCGCTCGCCTTCCCTTTCCGGCTCCCCCAGAATAATCCCTCCACGCAAAATAACGTATTGATAAAGCCTGACATCAAGTGAGAAATACGATAGAGAATCCCTTATAACTGTCAAATGCCGACCTGCGCAAGATGAGGATGCACTCCTCCATCAAGACACAAAAGAAACCACTTGGAGACGCTGACAGAGGTTCTGCGGCGAGGGTGAAACTGGACAATGCAGCAACAAGTGGCGTCAATGGGTCGCAAAAAAGGGGAGGTGATGAGGTCAAGTCGCACAGCCACACAAGCAAACAGCAGAAGTAAACCACCATCACATCTGAGCGGGGAATTTCGCTTTGATAAGGCATCCAGGCCGAGATCGGCAATCAGATGAATCATGTCAATCATCAGAAAAGTTCTTCCCCGCATCTTGAGGTATAAAACCGATGCATTGGACACCTTGAAACATCAGTCAGCTCCAGCAATCCAGTTCAGCAAC**ATG**CAGAACACAAGCATCCTAATCGTGGCCCTGGTGGCACTTTTCGCCATTACCGAGGCACTTCCCACAACAGGACCCATTCGCGTCCGTCGCCAGGTGCTCGGAGGTTCCTTAACCTCCAATCCCGCTGGTGGGGCTGATGCTCGTTTGGATCTGACCAAGGGCATTGGCAATCCCAACCACAATGTGGTGGGTCAGGTTTTCGCCGCCGGAAACACTCAAAGTGGTCCAGTCACAACTGGCGGAACTTTGGCCTACAACAAGTGAGTTCTCTAAACTAAGAGATTACTAGAGGATATATTAACTCTATTCTATTCATTTTTGAAGTGCTGGTCATGGTGCCTCTTTGACCAAAACACAC

Forward Primer: GGGGTACCGCCATCAGGCCACCACCCATTCTG

Reverse Primer: GGAAGATCTGTTGCTGAACTGGATTGCTGGAGCTGAC

> *Drosophila* Drs Promoter

CAATGAAAGTGATAATACGAATTGACCATGTAGCAATTTGTTTTGTGTCTATAGACTGAATTTTTTCCCCTACATTAATCAAAATTATATTTTTATATTTTATTTTGAGTTTACTTGGGTTTTTCATGAAATTAAAGATTAACCTGGGGTTTTTACAATCCATTACGATAGGCTTTCTGTTCATTGCATCTATAGCCTCTGTACTTTTCCGTGCATTCTTAAAGCAAAAGCATACATGTATATCTTCAATTCAAGTATATCTGCAATTTAGTTTGCTTATCTGGAGTCGCTGTATCCCGACCTATCCAAACTCGCGTCCCAGTCAAAGGTAAACCATTTTTATTTAGTTCCCAGCCTCTGGTATTTGTTGTTATTTATCGGTGACTTTTTGAAATTTATTCAATTAATTTCAGTCTTTGTGCTCTTGATAACACGATTTCCTCGTTATTCATTTAGTTTGGGTTTAACCAAAACCCTTTAGCGAATCATTTTTGCTGGACAGTCCAGTTGAATTCGGTATTCTACACACAAAGCTCATCTTACAGTGAAAAGTGTTACTTTATGAAATACAAATGAGGCTCTAAGCAATGCTTTTCGCTTACGCTTTTCGATAAGCGTACAAGTAGTTCCCCTACCGAAGGCCTATAAATGTGACTGCACATGTATCATCATAATTTGTTGATATACTTCGTTTATACCCGACTACGCATCGGCTAAAGCTGAGGGATCGGTGCACTATATAAGCTTCTCCTCGAAGTTCCCAAGCCACAAGTCGCTGATAATTCAAACAGAAATCATTTACCAAGCTCCGTGAGAACCTTTTCCAAT**ATG**ATGCAGATCAAGTACTTGTTCGCCCTCTTCGCTGTCCTGATGCTGGTGGTCCTGGGAGCCAACGAGGCCGATGCCGACTGCCTGTCCGGAAGATACAAGGGTCCCTGTGCCGTCTGGGACAACGAGACCTGTCGTCGTGTGTGCAAGGAGGAGGGACGCTCCAGTGGCCACTGCAGCCCCAGTCTGAAGTGCTGGTGCGAAGGATGCTAA

Forward Primer: GGGGTACCCAATGAAAGTGATAATACGAATTGACC

Reverse Primer: GGAAGATCTATTGGAAAAGGTTCTCACGGAG

>LvPEN4 Promoter

ACATGCAGATACAGATACATATATTCATATTTATATAATAAGTATGTATTTATCTACCCATGCCTTTATATTTACAATAATAGATGCCTATATGTATGCGAGTCAGAATAGAGGGCAACCATAACGGAAACACACAACGCTCATCGGGTCATTGCAATCTGTTTGAAAACTTCCCTAAAGACGATCGCTAACAGTGTGACTATTGTGTAGTTTTAGGTGATCTTCATTACGAGTGTTATTTATGTTTAGAAAATAAGGGGTTTTAGATATCAAAATTGATGATTAACAAGGATTCACTCGAAAAAATTATTTCATTTCTACCCTCTGTTCATTTGTTTATTTGTCTCATAGTCTGTTTAGCTATTTATTTCTCAGCCTGTCGTTCTGCTTAGCTACGTCACTTTATCTATCTGTCTCTCTTATCTCTCTTATCCCCCTTCTCTTTCTTTCTCCTACCCAGTTTCCCGTTTTCCCTTCCCTCCTATGCATCCTTTTTTCTCTCTCCCTTATGCCCTTTCGCTCTCTATATCTCTTCCATCCTGTCCTTTCTCTCTCCCAAGTTGCTTGCACAACCGCGCGGCGTCTCCATAAAAGGCATGGCACCGAAGCGTCCGGTGCCACTCGGCGCTTGGCTCTCCCTCGAGCCTCACCTGCAGAGACCGACGCTCCGAGCCCGGGTTGCCTCCTGCGTCCGCC**ATG**CGCCTCGTGGTCTGCCTGGTCTTCTTGGCCTCCTTCGCCCTGGTCTGCCAAGGGCACAGCAGCGGTTACACGCGCCCGTTACCCAAACCATCCCGACCTATTTTTATTCGACCGATTGGTAAAGCTTCTATAAGTTACTTGTTTGTTTATATGTTCATCAGAGTGCTATTAGTAATCACATGTTGATATCAGTAATCACCAACATGTCACATTGTATATTTATCACTATTATTGCAATCAATTTCTTCATTATTTGCAAATCTTTAAACCGTTATATTTTCTTTCTTTCTTTCCCAGGGTGCGATGTATGCTACGGAATTCCCTCCTCAACAGCTCGACTTTGCTGCTTCAGATACGGGGATTGTTGTCACAGAGGATAGTCTGGTTGATGGAGAAGACGATGAAAACCGGGCTTGAAAACGTCTTAATTCATACTTGTGTGTGAAGAGACTGTGATCCTGATTTTGCACTGTGTTTTCTCGTTCCATGTTCTTGATTTTGCTTGTGAAATGGACGTAGGCATTCGGTCTATGCTTTGCAAGGATTAGCTAAAGATTGTTCCATGAATGTACGGTGAATGAAAGCGCGCTTGGTATGTATGTGCTGCATCTAGTTTTATCTGTCCCAACAGTTGCTCCCGTATTCATCAA

Forward Primer: GGGGTACCACATGCAGATACAGATACATATATTCATATT

Reverse Primer: GGAAGATCTGCGGACGCAGGAGGCAAC

> PmPEN453 Promoter

ACCCCCTTTGAGAACTCTCCTGACAACGCTTACTAACAGCTTGCCTGTATAGTGTCAGATGGTCTCCATTACGTGTGGTATATGTTTAAAAAAAAAGGGAGGTTTAAAAGTTAAAATTGATGATGATGATGGTGAATAATGATGAAACTGAAAAATCTTATATTTTTTCCATGTTTTTTATCTGTCTGTCTGTTAATCTAATAGTTAGCTATCTATCTATTTACCATTCTGTTTAGTTTTGAGTCTCTTTTTCTATTTGTCTCTATGCCTATTTATATTTCTTTCTTTCCCTCTTACCCCCCTTCCCTCTCTCTCTCTCTCTCTCTCTCTCTCTCTCTCTCTCTCCTTTCTCTATCTATCTCTACCTTCCTGTCTTTCTCTCCCCCCGTCTCTCTCTTTAAAACTGCTTGCACAACCGCGTGGCGTCTCTATAAAAGCACCACAGCCCCCGGTGCCAGTCGGTGCTTGGCTCTCACCTGACCCCCACCTGTAGAGGCCGAGACTCCTTGCCCGGGTTCCTTCCTGTGTCCGCC**ATG**

Forward Primer: GGGGTACCACCCCCTTTGAGAACTCTCCTG

Reverse Primer: GGAAGATCTGGCGGACACAGGAAGGAACCCG

> PmPEN309 Promoter

CATTTACATGAAATTGAAAAGAACTGGACATCGTTTGAAAACCCACTAGATTCTCCCTCTTTTCTCGTTCTCTCTGTCTGCCTTCCTGTTTGTCTGTCTGTCTGTCTGTCTCTCTCTCTATATATGCTATATATGTATACATATGTATACTTCAAAGACCTTACACATCTCAATATATATGCATATATATGTGTGTGTGTGTGTGTGTGTGTGTGTGTGTGTGTGTGTGTGTGCATGTGTGTATAGATGTATGTAAAGCATGGAAAACGACTCAAGGTGCTTGCACAACATCGTGGCGTCTCTATATAAGCCAGCCACCTCAGCTTCCAGTACCAGTCGGTGCTTGGCTCTCACCTGACCCCCACCTGTAGAGGCCGAGACTCCTTGCCCGGGTTCCTTCCTGTGTCCGCC**ATG**

Forward Primer: GGGGTACCCATTTACATGAAATTGAAAAGAACTG

Reverse Primer: GGAAGATCTGGCGGACACAGGAAGGAAC

>WSSV069 Promoter

AGGGAGATCCTAGAAAGAGGAGTGAAGAAGGACGATAGAACTGGAACAGGAACTCTATCCATTTTTGGACCCCAAATGAGGTTCTCTCTTCGAGACGACACTATTCCAGTTCTCACTACCAAGAAAATTTTCTGGAGAGGAGTTGTGGAAGAACTCTTGTGGTTCATCAGGGGCAATACAGACGCCAAAGAATTGGCCAAGAAGAAGATACACATCTGGAACGCTAATGGGTCGCGGGAATTTTTGGACAGTAGAGGGTTATACGATAGAGCAGAGGGAGATTTGGGACCCGTATACGGATTCCAATGGCGTCATTTTGGTGCTGAATATGATACCTGTTCTTCCGATTATACTGGAAAGGGTATTGATCAATTGGCCAATATACTAAAGACCCTGAGAGAAAATCCAGATGATAGAAGGATGATTATGACGGCATGGAATCCTATGGATCTTCACCTTATGGCTCTTCCTCCATGCCACATGACTGCTCAATTTTATGTGGCTAATGGAGAATTGTCGTGCCAGTTGTATCAGCGAAGCGGAGATGTCGGGTTGGGCGTGCCCTTCAATATTGCATCATACTCTCTTCTGACTCATCTGATGGCCAGTATGGTGGGTCTAAAACCGGGAGAGTTTATCCTCACTCTTGGTGACGCACACATTTATAATACCCACATTGAGGTGTTAAAGAAGCAGTTGTGCCGCGTCCCTAGACCATTCCCTAAGTTGAGGATTTTAATGGCTCCAGAAAAAATTGAGGACTTTACTATCGACATGTTTTATCTTGAGGGGTATCAACCACACAGTGGAAACTTGCAGATGAAAATGGCTGTTTGAATCATGTTAAGGAATTTCCTTGTTACTCATTTATTCCTAGAAATGGTGTAATCGCTGTTGTGGGCGGAGCATATTTGTGTATATAAGAGCCCGTGTTAGCTCCTCGATTCAGTCACAAGAGCGCACACACACGCTTATAACTAGCTCTCTCTCTCCACTCAAG**ATG**GCCTTTAATTTTGAAGACTCTACAAATCTCTTTGCCAATATGGACTTGACGGCTGGCACAACAACAGACCCTACCCGCCCCAATATCATATTCTTTGAAAGTCTACTCCCCAACTCTGGTATTGAGGTGATGAAGAGGCGTCTCGTACGGCAAGGAAAGTGTGGGAATTTTGAAGCAAGTGGAGGTGCTATGTCGTATTTCTGGCTCGAAGATAATGCAGAAGATATGGAGAATCTCAACAGTGGTTCCCATGTCAAGACAAACTGCTTGGCATTATTCCTTCAAGAGTTTATCAGCAACTGGATTGAAGAGACTGATCGACATGGACAGTACTGTACTTTTCCCCAATACATGGACGGTGGGGATGGTTCACGTGGGGGATATTTTACTTCGCTAGCCATGAAATGGATGGCTAGGGATGTGACTTTCTTTGTGTTTGTTGATAGGAATAATACTGTAGAAAATGCGGCATCCATATGGATGTACCAAAAACTACTAGCAATTGGTGCAAAGGTAGTAAAGGTGATTGTTGACAATGCATCAAACCCAATGTTTTCTGTATGTAATGCGTGTAGGTGCAAGTACCCAGGCCCAGTGTCATACGTTATTGAAGGCCATGGAGTGGGTCATTCTGATTTGACATGTGATGAGATTTCTGGATTCTTTGTATAA

Forward Primer: GGGGTACCAGGGAGATCCTAGAAAGAGGAGTG

Reverse Primer: CCGCTCGAGCTTGAGTGGAGAGAGAGAGCTAGTTAT

>WSSV303 Promoter

GTTCAGAGATGTCCAGGTCCTTATCTGGAGTGTTGAACCGGACGTGGCTGATGAAACTGGATTCGGTGTTGCTCCCGTTCCTGTTCTTCTTATTCTTATTCTTCTCCCACGGTGAGCAAGAAATGCAAGTAGTAGTTCTGTCGATGCACTTCACGAGCATGTTCATGGCCAGTGCGAGCCCAATAGTCATGGAGCAGCCGACTGCCAATCCACTCAGGAAAGTGACGAGGATAATGTTGTCGTTGGTGATAAGATTGTCCATCTCGAGAGATGTTTCTTCCTCGACTACCAGACGAGATATGATGCTCTGGGGCCCTGCTTCGTCCTTTTATACCGTCACTTGGTTAGAAAAAACGATAGTGTAGTTCACAGTATTTGTCCCTTCTGGTCTTGGGATTGGGGTTCTGATACTCTGCCCAGCCTCCAGATATAGGTGGATAGACCGAGATTCTTCCCTTGATGCAGTTTCTATTCCATCCAAGATATCTTCACCAAATAAGTCCTTATGCAAGGACACCAGAATATTGAACTCGTGAACGCTTGACTTGTTGATGATGCATAATTCACCTTGTTCGACTGTTATACATACTCCTTCAATAGAATCGTATAGCCCGGGGTTATTGGCACGGTCCTCTTCAGGTAAGAAAACTGCAAATATTGTATTAGGCACAACATCTGGCACGTTTACAGAATGACAGTAATGTTTATCTATAATGTCACCAGAAAAGTGAAAATCTATCATCCGACACTGGAGAAAGGTTGAAGGAAAATCTTTCCGCATATTCTTGAGTTCCCGCAATAACTTCTTGTTTCTGGAGTTTGACTTTTTAGGATTATTTGTTGCCAGACTTACAGCATCGAAAATACTTTCTCCCATTCCTTCAGCGGGATTTTCCATCTTACTTTTGTAGTGGTTTTCAGTTCATTAATTGTGTTTCAGGGAGGCTATAAAACAAAACTCTTACGCATCTGTTCATTCAGTAGTGGACCAGACGTCGAG**ATG**ACGAGACATGGTGTGCTTGTTCCAAAAGGCCGTTCTAGGCATGTTATCCTAGGAAATGTCGACTACACTTTCTGTACTACTGACAATAATTGTGTCAGTCTAGACATCGATTTCAAGGACAATATCACAGACCAAAACATTCAGTTATTGAACAAGAAATTGGGTAAGAAAACAGCAAAGAAAATAAAGAAGGAAGATGCACCTGAAACAAAGGAAAATAGTGACGAAGACATATACGCCACCAAGGAATTCGAACAGACAATAAAAGGTCTACAGACAAAAAAAGGTGCCACCGAGGAAAACGCCATCGCGGCCGCAGCTGCCGCTGCCACTGCTGCTGCGGTAGAAAAGGCTATGCTATCAGAAAGTGAAGGAAAATCAATGGTCATCAACAGAGCTAGAATGGTGCTTTCTAAGCGAGACACGTCCCAGAAACAGTTCACTGCATTGAAGAACAGGGAATCTTTCTTCAGTGTTTTGATATTTGAAACTGGATCAGTGATAGTTGTCGGGCTTCAAGATCCTTCGCTTACAAAATTGTGTGTGATTAAAGCCACGACTGATATTGCTGATATTCTACAGAAAAACATCAGTGTGGCTAACGTGTCTATAGTGAATACAGTGTCCACTTTTAATAGATTCCACTTGAACTTTATTCGACTCGGGAAATTCTTCGAAAGAAATTGCATCTCTTACAGTTATAACCCAGAAACGTTCCCCGGTATGTTTTTCAAGCTGCGAGTGCCCGCAAAGCCTCTCTTGCCTGGAGAGACTATAGGGGAATACTACACAAAGGTTGCAATGATGCGCGATAGTAAGGATCCCAATTTTAAAATGTCTGACTGGTTGAGGATAAAAACTGCATTAACATTTAAAGTTGGGAAAATTACTGTGCTCGGAGAAGGAGAGAGTGGGTGCGGTGATGTTTCTGTCGTATCCAAATTACTATTTGGTTTATTCCATTACTTTATGGACAACAACATTAAAATGTCCCCCAAAGAAGCACAAAGAGTCAGAGAAAAATACGGCATCCCGCATCTAGAATGGTACTTGTACATTGACATGTTGCTCCACTCCTACCCGTACGTCAAACCATCGGCCGAGCAAGTGAAAAGGGCGATGGTGGACCAACAACATATTTCTGAAGTGGATAGGACATACTATGGAACAAAGAACAGTATGGACGCTGCCATGTCTGCAAATTTAGTGCCTTCAAAAGAAGAGAGTATCTCCTTCATTAAAAAAATAAGATCACAACAACTTTTTGGACATTTGTGTAAACCTTCAAAAGAAACTACTCGACGTGCTATAGACACACTTTCTTTCGATCCTATAAACCAAGACAGGTGGTGGAATAAAAATGACCAATATTACGGTAAAGAGAGATGTGACCCGTTTTCTGTTGCACGTTTAGTGTCTGTTTCTGAAAATACAAACAGTATGATGAATAGTCGCATTTCTTGTCAAGGGAAATGGTGGCTAGATGAAAACGAATACAAGGATAAACTTGATCATATTGTGGATTTGTGTACAGAAGAAATAGTGGAGGAATGTGAATCAAAGGGTTTTATTGCCTCCCCATTTTTGAGGAAGCACCAGAAGGAAAAAATACCAACGCCTTATGTTTTATTAGCGAGAGCCTGTAATCAAAAAAATGGTAACAAAATGAGTATTAACAATAATAGTAACTATTTGTCGGGTTCAAGTAGGGCGAAGAGGAATGCAAAACTACAGGAAAAACACCGTGTAACTTTAGCCAGGTTGAACACGATGATGGCGTCGTACCGATTTTTGAATAATTACATCTCAACAGACATTGCACCTGATTTTGCCAAGTTATTTGGTAATGATGTATATAGTTTATTACATTTAATGACAAACTTGCCTAAATCCCGTGGACATGCTCTAACATACAACGAAAGGGCCCTTTCAAGTAATGAAAGTACATATAAAACACCTGGAAATGCATACTTTAGTACTCTATTTGAAAAATCCATTATAAATAACCAAGAAACTGCTAATAAAGGTAACAATAGAAAACGTAAATTTTCTCGAATCGGACAAGAAAAGAGCTCTTTTCTGTGCAACGCGTGTGGTGTCAATTTGAACAAGGGTAGTGATGAAATCATAAAGGGTATTTGTACAAGTTGCGATCAAAATAGTACCAGTTACATAGAGAATGCATTATCTGACATTAACAGAGACAAGAAGATTAAACGTTTTAAAGCAGCTGCAACCCATCCGCCAGTGAAGCAAGAATTGGTAGATTCTTTATCCTCCTCTTCATCTCCTTCTTCTTCTTCTTCTCAGACGTCTAACAAGAACAATAGATGCACCCCTAGTGATTTTATAGATTATGTGTACAAATTCACTGACGAAACAACAGGTGCTCCAAAGGTGGGCTTAGTGTTTAAAATGTGTGATATTCTTGCATCCTTAGCAAGCAGGAGAGGGATGGAAGATCGTCCCACAGCCAACTATAGAACCTCCTTACATTCAGCTACTCAAAATAAAACCAATTTGAATAAACTATTAGTTTCTGCTATCAAGGAAACAGGAGCCACTGAAACTGAAGCACAGATATTCAACAAGATTATTGGTAGTGAAAAGGGACTATCAATTCTCTGTCAACTTGTGGAAAGGAGGAACAAAGACAATAATGTCTTCGACTGA

Forward Primer: GGGGTACCGTTCAGAGATGTCCAGGTCCTTATC

Reverse Primer: GGAAGATCTCTCGACGTCTGGTCCACTACTG

>WSSV371 Promoter

GTGCTTGGTGGTAACAGAAAGTACGTTACTCAAATCGTTAGATTCTTGTGTTTGAAGTTGTTGGAGAGCCACTTTTTTGTGCTCAACATTATCAATATTGGCAGATGAAGAAGAAGATGATGATGGTGATGAGGATGATGCGTCAGAAGAATTGACATTAGATTGTTTGTTTTGGAAGACGATAGGCATGTAGGGGATAGTTTTGGCAAGGTTGTTTGAATAGTCCATTGAGAAAGAGGAAGATGATGATGATGGGGAAGAACTGGGAGAAGAAGAGGAGGATGTTGTGTTAGATGATGAGGAAGAAGAAATGGCCGAGCTAGAAGGAGATGCCTTCTCTGCGAGTGCTTCGCCTAGTTTTTCAATGAGTTTGTCCATATTCTTAATATCGGCATCATCAAGTGCAAAAATGTCGTTCATGGCATATTTTCCAAAGCAAAGAGACTGTAGACGAGAACGGATGGTTGCATTTCTATTGTCCTCTACAATTGCGAGGATGGTTTTAGCTACTCTACGCTGATCATTGTCCTTGATAGAACTGGTGTTATTTTCTTCATCGGTCTTCAGCCAGATACCTGTTTCTTTGTGCTTTATGAATGTACCACCTTGGTCGTTAGTCATTGGTACAAAGTTACTATTTCCTTCAGTCAATTTCTTACATTCGAGGTTATTTTGGGCGGACATACGGTTGATGTCGGCTGAGGCAACGAGCAGGCTCAACATTTTTTCGTGCAGTTGGGATGAACCTTCAATTGCGGCGGTCACGTGACGTTTTGCCTCTTCAATGTTTTTCAGTTCATTTTGAACAATAAATTTGAGGAGAGTATTGTCTGTCAATAGTTCACTAACAGATGCTCCTCTCTCGCTGGTGTCATCCGCATACACGGGGGTTAAAGATTCAGCAATTACGGCAACGACTGCGCGTGTCTCGGAAAGTCCATCATTACTAATATGGCTGAATTGTTTTAGTTTGTTGTAGTGTTCGGCGAGTCTATTCTCA**ATG**GTTCTCAGATTGGCCTTTGTATTTGAAAGAACAGCAGATGTTTCTAGTATCCTTTCAAATTCAGATGTGGAAATCTTGGCCAAAGTAGAACCACTGCGGGATGAGAACGAGTTGATTCCCCTTGACTTGGAAATGTCCATCACGATAGTGCTAATAGAGTTACGCATTTCCTTGGCTCTAACTACAATTGCTTCAGAGGAGGATGGGAAGTTCTTATAG

Forward Primer: GGGGTACCGTGCTTGGTGGTAACAGAAAGTACG

Reverse Primer: CCGCTCGAGTGAGAATAGACTCGCCGAACACTAC
