# Supplementary material for: Analysis of Expression, Cellular Localization, and Function of Three Inhibitors of Apoptosis (IAPs) from Litopenaeus vannamei during WSSV Infection and in Regulation of Antimicrobial Peptide Genes (AMPs)
Source: PLoS One. 2013 Aug 14;8(8):e72592. doi: 10.1371/journal.pone.0072592 (PMC3743791; doi:10.1371/journal.pone.0072592)
Supplement: Figure S2 — cDNA sequences of WSSV VP28 and Litopenaeusvannamei AMPs including LvPEN2-4, Lvlysozyme, Lvcrustin1-3, LvALF1-3, and LvVICP1-2. The qPCR primers are also provided and underlined in the cDNA sequences. The ORFs of AMPs were shaded. (DOCX) [file pone.0072592.s002.docx]

Fig.S2. The nucleic acid sequences of WSSV VP28 and various *L. vannamei* AMPs used in this study. The qPCR primers are also provided and underlined in the nucleic acid sequences. The ORFs of AMPs were shaded.

> WSSV VP28

ATGGATCTTTCTTTCACTCTTTCGGTCGTGTCGGCCATCCTCGCCATCACTGCTGTGATTGCTGTATTTATTGTGATTTTTAGGTATCACAACACTGTGACCAAGACCATCGAAACCCACACAGACAATATCGAGACAAACATGGATGAAAACCTCCGCATTCCTGTGACTGCTGAGGTTGGATCAGGCTACTTCAAGATGACTGATGTGTCCTTTGACAGCGACACCTTGGGCAAAATCAAGATCCGCAATGGAAAGTCTGATGCACAGATGAAGGAAGAAGATGCGGATCTTGTCATCACTCCCGTGGAGGGCCGAGCACTCGAAGTGACTGTGGGGCAGAATCTCACCTTTGAGGGAACATTCAAGGTGTGGAACAACACATCAAGAAAGATCAACATCACTGGTATGCAGATGGTGCCAAAGATTAACCCATCAAAGGCCTTTGTCGGTAGCTCCAACACCTCCTCCTTCACCCCCGTCTCTATTGATGAGGATGAAGTTGGCACCTTTGTGTGTGGTACCACCTTTGGCGCACCAATTGCAGCTACCGCCGGTGGAAATCTTTTCGACATGTACGTGCACGTCACCTACTCTGGCACTGAGACCGAGTAA

QPCR-VP28F

AAGACCATCGAAACCCACAC

QPCR-VP28R

TCGCTGTCAAAGGACACATC

>LvPEN2

GGCCACTCGGCGCTTGGCTCTCCCTCGAGCCTCACCTGCAGAGACCGACGCTCCGAGCCCGGGTTCCCTCCTGCGTCCGCCATGCGCCTCGTGGTCTGCCTGGTCTTCTTGGCCTCCTTCGCCCTGGTCTGCCAAGGCGAAGCGTACAGGGGCGGTTACACAGGCCCGATACCCAGGCCACCACCCATTGGAAGACCACCGCTCAGACCTGTTTGCAATGCATGCTACAGACTTTCCGTCTCAGATGCTCGCAATTGCTGCATCAAGTTCGGAAGCTGTTGTCACTTAGTAAAAGGATAAAGAAATTGACGGAGAAGACAATGGAAACCTGGCTTGACTTGTTAATTAATACTCATATGTGAAGAGATTGCAACCCTGATTTTGAGCTGTATTTTCTCGTTCAATTTTGTTTACTTTTGCTTGTGGAAAGGATGTGGGTATTTCGTCTATCCATCGCTAAAGATTTTTCCATGAATGTATGATGAAGGAAAGTGCATGTGTGTAAGTATGTATGTATGTGCTTACAGGTATTTGTTGCATTAAGTGTCCGTGTATTTAGGATTTGCAACACACGAGGAAGAGAATATTTGCCACTTCCCATTTATTTCAGTTTCTGTAAGTATGGATCTGTGAGAGGTTGGTGTTGACAGATCTCTCTTTTACAAATAAAGTTGATATCTGTAAG

QPCR-LvPEN2F

GCATCAAGTTCGGAAGCTGT

QPCR-LvPEN2R

ACCCACATCCTTTCCACAAG

>LvPEN3

GGCCACTCGGCGCTTGGCTCTCCCTCGAGCCTCACCTGCAGAGACCGACGCTCCGAGCCCGGGTTCCCTCCTGCGTCCGCCATGCGCCTCGTGGTCTGCCTGGTCTTCTTGGCCTCCTTCGCCCTGGTCTGCCAAGGCCAAGTGTACAAGGGCGGTTACACGCGCCCGATACCCAGGCCACCACCCTTCGTGAGACCTTTGCCAGGAGGGCCTATTGGTCCATACAACGGTTGCCCTGTCTCATGCCGGGGAATTTCCTTCTCACAAGCGCGTTCTTGCTGCTCCCGGTTAGGGCGTTGCTGTCACGTGGGAAAGGGATATTCCGGTTGATGGAGAACACGATGAAAACCTCGCTTGACAACCTGTTGATTGATACTTGTATGTGAAGAGACTGTGATCCTGATTTTGCACTGTGTTTTCTCGTTCAATATTCTTACTCTGGCTTGTGGAATGGATGTAGTTATTTGACCCTATGTTTTTTTTTTTTTTTTTAAGATTTTTCCATGAATGCACGATGAATGAAAGCTTGCGTGATATGAGTGAGTGCATCCACTTTCCAACGTCCCAGCAGGTGGCGCCGTATTCATGATTTGTGACACACGAGGAAGTGAATCCATGCCATCTGCCTTTCGTTGTAATTTTTAGTGAGTATGGATCTGTGTGTGGTTGATTTTTACAAATCTCTCAAAGGACTTTTAGAAATGTTACTCCTTTACAAATAAAATTGGTATCTTG

QPCR-LvPEN3F

CTCTGGCTTGTGGAATGGAT

QPCR-LvPEN3R

GCATGGATTCACTTCCTCGT

>LvPEN4

GGGGGCCACTCGGCGCTTGGCTCTCCCTCGAGCCTCACCTGCAGAGACCGACGCTCCGAGCCCGGGTTGCCTCCTGCGTCCGCCATGCGCCTCGTGGTCTGCCTGGTCTTCTTGGCCTCCTTCGCCCTGGTCTGCCAAGGGCACAGCAGCGGTTACACGCGCCCGTTACCCAAACCATCCCGACCTATTTTTATTCGACCGATTGGGTGCGATGTATGCTACGGAATTCCCTCCTCAACAGCTCGACTTTGCTGCTTCAGATACGGGGATTGTTGTCACAGAGGATAGTCTGGCTGATGGAGAAGACGATGAAAACCGGGCTTGAAAACGTCTTAATTCATACTTGTATGTGAAGAGACTGCGATCCTGATTTTGCACTGTGTTTTCTCGTTCCATGTTCTTGATTTTGCTTGTGAAATGGACGTAGGCATTCGGTCTATGCGTTGCAAGGATTAGCTAAAGATTTTTCCATGATTGTACGGTGAATGAAAGCGCGCTTGGTATGTATGTGTTGCATCTAGTTTTATCTGTCCCAACAGTTGCTCCCGTATTCATCATTTGTAACATACGAGGGAGAGACTACTTGTCTTTTGCCTTTCGTTGTAGTTTTCAGTAAATATGGATCTGTGTGGTTGTTTTTTACAAATCTCCCAAAGGACATCTAGAAGTGCTACTCTTAAAAATAAAATTTCTTTCTGG

QPCR-LvPEN4F

ATGCTACGGAATTCCCTCCT

QPCR-LvPEN4R

ATCCTTGCAACGCATAGACC

>LvALF1

AGTCGGCGACGGACAGGCTTCCGAGCAACACCGCTTCCGCATTCGGCCTTGACTTCGGGGGGAAAAGACGACGATGCGGGTGCTGGTCAGCTCTGTAGTGGCACTCGCCCTGATTGCTCTTGTGCCACGGAGCCAGGGTCAGGGAGTGCAGGACCTCATCCCTTCGCTAGTCCAGAGGATAGTCGGGTTGTGGCACTCGGATGAGGTGGAGTTCATGGGTCACAGCTGCAGGTACAGTCAGCGGCCCTCCTTCTATAGGTGGGAGCTTTACTTCAATGGCAGGATGTGGTGTCCTGGATGGGCTCCCTTCACTGGCAGATCTCGCACCCGCAGCCCTTCCGGCGCCGTCGAGCACGCGACGAGGGACTTCGTGCAGAAGGCGCTGCAGAGTAATCTCATCACGGAGGACGACGCTAGAATTTGGCTCGAGCACTAAGGCCTTTGTCTCAAGGTCATTCCCATTTCCTTTTGACGCGATGAAGGTCGAAGCGATATTTGTAAATCGTGAATAAGAAGAATGATGTCAGC

QPCR-LvALF1F

ATAGTCGGGTTGTGGCACTC

QPCR-LvALF1R

GTCGTCCTCCGTGATGAGAT

>LvALF2

ATTGTCTGCTGTTAACTTGAGAGTAACTTTCCTAGTTTAGAGGATGCGTGTCTCCGTGTTGACAAGCCTGGTGGTGGCGGTGTTCCTGGTGGCACTCTTCGCCCCAGAGTGCCAGGCGCAAGGATGGCAGGCTGTGGCAGCGGCCGTCGCCAGCAAGATCGTTGGGCTGTGGAGGAACGAGGAGACGGAGCTGCTGGGGCATAAGTGCCGCTTCACCGTCAAACCTTACATCAAGAGGTTACAGCTGAACTACAAGGGGAAAATGTGGTGCCCCGGCTGGACGACTATCAAAGGGGAAGCCAGGACACGCAGCCATTCCGGGGTGGCTGGAAGGACGGCCAGGGACTTCGTCGAGAAAGCCTTCAGGGATGGCCTCATCTCCGAACAAGATGCTAAGCGGTGGCTGAACTAACAGGCCCTCTCCTGCGTGAAGAGCTGTCGGGGTTCGAGCCCTCGTTGGCAGTGGAAGCTCTGCCATCTTGAGCTGTTGTGTCTCTCTCTTCCACGTAGAGTTGACGTCTTGAGCTATTGTTGTTGTCGGATTTTTTGTTTTACTTAACAACTACACGAAAGTATATAACAAAAGCCGGTAATTGACGGTCTTAGAAGGATCCTCAGACTCTTTTGTTATTGATATTAAGGCAAAGTAGATTCTTTGAAATGTGACTTTAATAAACTTGATCCTTTTTAAAAAAAAAAAAAAAAAAAAAA

QPCR-LvALF2F

CTGTGGAGGAACGAGGAGAC

QPCR-LvALF2R

CCACCGCTTAGCATCTTGTT

>LvALF3

GAGACTCGGCAATTCTAACTGCTGAACTGAACTCCTCGGGACACTGTGGCAGCCGGAACAGCGTCAAGCAGACAGTCAGCGTTAAGAGAGTGGTTTCCCGTCCTTCAGGAACTTCGATTCATAACACTTTCAAGATGCGAGTGTCTGTCCTCAGCATGGCCCTCGTGGTGGCGTTGGCTGCGTCCTTCGCGCCGCAGTGCCAAGCGAGTGGCTGGGAGGCGCTGGTGCCGGCCATTGCGAACAAACTCACTGGACTGTGGGAGAGCGGAGAGCTGGAGCTGTTAGGACACTACTGCAACTTTAGCGTGACACCGAAATTCAAGCGCTGGCAACTGTATTTCAGGGGTCGCATGTGGTGCCCAGGATGGACAGCCATCAGAGGCCAAGCCGAGACCCGTAGCAGATCGGGCGTGGTAGGCAGAACTACACAGGACTTCGTCAGGAAAGCTTTCAGCGCGGGTCTCATCACCGAATCAGAGGCCCAAGTTTGGCTTAACAGTTAAGGCGAAGAAGAACGACACGCAGATATAATTTATAAGAGCGCTATGGAAGGGATCTCATTAATGGTAAACCCAAATTCTTCCGCGAATGTTGATACCTCATCCAATAAACATTAATATAATG

QPCR-LvALF3F

GATGCGAGTGTCTGTCCTCA

QPCR-LvALF3R

TCGGTGTCACGCTAAAGTTG

>Lvlysozyme

TCTTTCAGCTGAACAACAAGTACTGGTGCGGCGACGAGTTTGGCAAGAACGTGTGCGGAATTCCGTGTTCGGCCCTCCTGGACGACGACTTAACAGACGACCTTGCGTGTGCCAGAAAGGTCATTAAAGACACCGAACGATGGAAGGGCAAAGGAGAAGGCCTGACCGCATGGGTCGCCTACGTTAACAGATGTCAGAACCGAAACTTGGACGAGTACATATCGGAATGCTGGACAGGTGATGCGACGGGTTCCAACATCATCAACATAAAGAACGAGTCCCCCATCGAATCAGCTGACACAGAAAACGAGGAGGTAGTCGGCGTCAGTGCTGGGGGTGACTCGCCTATCATCAATAATGTTAGAGTCCCCATTCAGTATCAAGTGTTGCCTATTTTTCAGCCTGTTCCACACGTTGCTTATTCATCTCCTATTGTGATGAGGAATCCCTATGGATGTATTTACCAGCATGCTCTCCAGCAATAGGTCAGATCTGCTGACCGGAGAAGGCATTTGCGAATTTAGGTGGATGAACACATTGCATGAATTATATGCAGCGCGTAATTCATTCACGTTTGTTTATGTGTTTGTTGTTATTCATATTTGCGTTATGTATTTATTCATGCACTATCATGGTAGTTTGCAAACCTTTTGCTGTCAATATTCACAAATCAATAAAGATATAACAATAAAAAAAAAAAAAAAAAAAAAAAAAAAAAAAAAAAAAAAA

QPCR-LvlysozymeF

AAGACACCGAACGATGGAAG

QPCR-LvlysozymeR

TGGGGGACTCGTTCTTTATG

> Lvcrustin1

GCATAGTTGCTGTCCTTCCAACGACCATGAAGGGCGTCAAGCTGGTGGTCCTGTGCTGCCTTCTGGTGGCCGTTTCGGGTCAAAGGGGCGGAAGRCAGCGAGGTCAAGGCAGATTCTTCCAAGGGGGCGGCGGTGGCTTCCCCGGGGGCGGTGGTTTCCCTGGCGGTGGCTTCCCCGGGGCGGTGGCTTCCCTGGCGTCGGCGGTGGGTTCCCGTCGCAGTGCAGGTACTGGTGCCGCACGCCCGAGGGCCAGGCCTACTGCTGCGAGGGCGCCCAGCAGCCGGAGCGCCCTGTCGGGACCAAGTTCGGGCTGTGTCCTCCKGTACGACCCACCTGCCCCAACACCCGCTTCGGCCCCCCCCAGACGTGCTCCAACGACTACAACTGCGCCGGCAGTGACAAGTGCTGCTTCGACAGGTGCCTGGGCGAACACGTGTGCAAGCCGCCCTCGATCTTCGGCCAGTTCGGATAAAACGAGAATCGGGGAATTATCTGTTCGATAATGGACGGTCAATAAAGATGGCGGATGAGACCACATCCAATCT

QPCRLvcrustin1F

GTCGCAGTGCAGGTACTGGT

QPCRLvcrustin1R

TAGTCGTTGGAGCACGTCTG

> Lvcrustin2

ACACAACTATCCCTCACATACACCATGAAGGGTCTCAGCGTTATCCTGTGCTGCGTTCTGGCGCTGGTGTCAGCCAATGGCAAGGGGAATCAGCAGGGGAACAAGAGATTCTTTCAAGGAGGTGGTTTCCCTGGGGGACATGGCGGTTTCCCCGGAGGGGTCGGTGGCGGTTTCCCCGGAGGGATCGGTGGCGGTTTCCCCGGAGGGATCGGTGGCGGTTTCCCCGGAGGGATCGGTGGCGGTTTCCCCGGAGGCTTCCCCAGCGCCACAGCCCCTCCCGCCACATGCAGGCGCTGGTGCCGAACTCCAGAGCAACAAGTCTATTGCTGCGAGTCCGCCTTTGAACCCGAGGCCCCCGTGGGCACCAAGCCCCTCGACTGCCCACAAGTCCGTCCCACCTGCCCACCCACACGCTTCGGTGGACAACCTGTAACCTGCTCCAGCGACTACAAGTGCGGCGGCCTGGACAAGTGCTGCTTCGACAGGTGTCTGGCAGAACACGTGTGCAAGCCCCCTTCCTTCTACAGCCAGTTCGGTTGAGAAGGAAAATTCACCGTGCTGATTCATGTAATGTCAATGATTAATAAATGTTTTCAA

QPCR-Lvcrustin2F

ATCAGCAGGGGAACAAGAGA

QPCR-Lvcrustin2R

CGGACTCGCAGCAATAGACT

> Lvcrustin3

ACAGCAGTTAGGTGCCTACACTGATCCCCCAAAGATGAAGAGACTTTTGTTGCTGGTCGCGATTTTCGCCCTCGTGGTTGCTGACGACGACAATGACGCAACAGATGGCGTCGAGGGTCGTTCCAGAAGCAAAGCTGAGTCGGGCAAGAAGGAATCCAGATTCTTTGGAGGATTAGGAGGTTTTGATCCCGTTGGAGGATTGGGAGGAGGATTTGGATTGGGTGGAGGTCTTGGAGGATTGGATGGAGGATTTGGAGTTAACCCAACACTTGGAGGAGGATTCGGATTCAACCCAGCTCTTGCGGGCGGATTTGGAGTAAACCCTGCCTTCGGAGGAGTAAATCCTGCATTCACTCCTGTGGCTCCTCCCTCCACATGCCGTTACTGGTGCAGGACACCCGAAGGCCAGGCCTACTGCTGTGAGAACATCAACCAGCCACAGAGTGCTGCCGGTGTAGTCAAACCGGGATTCTGTCCCCCCGTTCGTCCAGTGTGTCCTCTTAGGAGCTTCCAGCCACCATTCACTTGCTCTAACGACGGCGCTTGCGGAGGCATCGACAAGTGTTGCTTCGACAGATGTCTCGGCGAGCACGTGTGCAAGGCTCCTCTGGGCATTGGGCGATAGATATCCTTGGGAAAATTAAGCTGTTCAGTGAAATTCATCATTGCCTTTTCGAGGTAGGATGATTTCATGAACATTAATGTTTTCGTCCATTGAAACGTATGTAAAAAAGATGTAAATACAATG

QPCR-Lvcrustin3F

GACAATGACGCAACAGATGG

QPCR-Lvcrustin3R

CTCCATCCAATCCTCCAAGA

>LvVICP1

GGGGTCTTCATTCGGGCTCCTGTGTGGTGGCCGAACTCTGGACGCATCCCTGCTGCCATCATGAAGACCTACAGTCAGGTCTCTGTTTTTGTCTTATTGGTTGCGATCGCGCACACGTCACAAGGATCTTCCTTTTCACCACCTAGAGGACCTCCGGGCTGGAAACTTCCATGCGTACCACAAGAGTGCCCACCGTGCCCATATGATGATTATGAGTGTCCGAAGTGCGGTGGATTCCCGGTGTGTCACGAGGTGTGCACCGATATTAGTATATCATGTGAATGCGGCTACCATAGCTGCGAATGTCCGCGGCCTGTGTGTGAGCCGTGCGAAAGTCCCATCGCCGAGTTGATCAAAAAGGGAGGCTATAAAGGATAAAGAGAACGAAGGCGCAGCATCCCTTCTGAACTGAAGAGAGAGAGAGAGAGAGAGAGAGAGAGAGAGAGAGAGAGAGAGAGAGTAGATAGTAAGATGGGAAAAAAGGAGAAATGTTTCTCAAAATAGTTCAAGAATCAACAAATTACTTCGACGGTGACTGTTTTTTTTCTTACTATTTTTCTCTTTATGTTAACGGTCCAGCACCGCAAGATTTAGCCATTGTTCTCTTGATGGCAATTCTGTAATTAATGTTCAGTCAGTGGTTCTTCCTTCTGTCGTGTCGCTGATTCAAATTATCAGATTTTATGTGTGGCTGTTCCAGAAGATCAATAAATATTCTTTGTATTTGTATACACCATTTCGACTATGTACATTATGTGTTTTGTCTCAGTCAATAAAACAGATTTTCGTTTAAAAAAAAAAAAAA

QPCR-LvVICP1F

ACCGTGCCCATATGATGATT

QPCR-LvVICP1R

TGCGCCTTCGTTCTCTTTAT

>LvVICP2

ATTCGGAGTCCAGTCGCTGTACTGCTCCTGTGTAGTAGCCGAACTCTGGATACATCCCTGCTGCCATCATGAAGACCTACAGTCAGGTCTCTGTTTTTGTCTTATTGGTTGCGATCGCGCACACGTCACAAGGATCTTCCTTTTCACCACCCGGTCGACTTCCGGGCTGGGAACCTCCATGCGTACCACAAGAGTGCCCACCGTGCCCATATGATGATGAGTGTCCGAAGTGCGAGGAATTACCGGCGTGTGAGGAGTGCCCCGATATTCATATAGGATGTGACTGCCCTTTCTACCACAGCTGCTTATGTCGGCAGCCTGTGTGTGAGCCGTGCGAAAGTCCCATCGCCGAGTTGATCAAAAAGGGAGGCTATAAAGGATAAAGAAGCGAGAACGAAGGCACAACATCCCTTCACAAACGTCACTGAACTGAAAGGAGAGAGAGAGAGAGAGAGAGAGAGAGAGAGAGAGAGAGAGAGAGAGAGAGAGAGAGAGAGAGAGAGAGAGAGAGAGACGTTAAATAGTAAATAAGATGGGAAAAAGGAGATAAGTTTCCAAAAATCGTGCAAGGAAGAACAAAAAACTTCGACGCTCAATGGTTCTTCTATAACGATTCCTCACTATATAAGTTGATGGTCCATCGCTGCAAGACCTAGTCTCGGATCTAGCCATAGTACTCATGATGGTAATTCTGTAATCAATGTTCAGTTGGTGGTTCTGCATTCTCGTTTTATTGATTTTTTTTTTTGTGTGTGCGGCTGTTTCAAGTGTTCTTCTATAAGTATTCTTAATATTTGTATATGCCATTTTGACTATGTGCGAATGTATTATCTGTGTTAATAAAACAGATTTTCGCTG

QPCR-LvVICP2F

AAGGCACAACATCCCTTCAC

QPCR-LvVICP2R

CCATTGAGCGTCGAAGTTTT
